# The Mechanism of a Novel Mitochondrial-Targeted Icaritin Derivative in Regulating Apoptosis of BEL-7402 Cells Based on the SIRT3 and CypD-Mediated ROS/p38 MAPK Signaling Pathway

**DOI:** 10.3390/molecules30081667

**Published:** 2025-04-08

**Authors:** Zenan Chen, Wei Li, Yan Zhao, Dingrui Liu, Jiahong Han, Enbo Cai

**Affiliations:** College of Chinese Medicinal Material, Jilin Agricultural University, 2888 Xincheng Street, Changchun 130118, China; chenzn1999@126.com (Z.C.); liwei7727@126.com (W.L.); zhaoyan@jlau.edu.cn (Y.Z.); ldrui1113@163.com (D.L.)

**Keywords:** icaritin, mitochondrial targeting, SIRT3, CypD, apotosis, p38 MAPK

## Abstract

Tumorigenesis and progression are closely associated with apoptosis and primarily regulated by mitochondria, which are considered major targets for cancer therapy. In this study, twelve novel icaritin (ICT) derivatives were designed and synthesized, four of which were specifically targeted to mitochondria. Biological studies demonstrated that all compounds containing triphenylphosphine (TPP^+^) exhibited a substantial increase in antitumor activity compared to ICT and control compounds while also exhibiting notable selectivity for tumor cells over normal cells. Among these derivatives, Mito-ICT-4 exhibited the strongest antiproliferative effect, with an IC_50_ value of 0.73 ± 0.06 μM for BEL-7402 cells, which is 29 times lower than that of ICT, and an IC_50_ value of 67.11 ± 2.09 μM for HEK293 cells, indicating approximately 33-fold selectivity for tumor cells. High-performance liquid chromatography (HPLC) analysis revealed that Mito-ICT-4 significantly accumulated in the mitochondria of BEL-7402 cells, with the level of accumulation approximately 2.5 times greater than that of ICT. Further investigations demonstrated that upon entering the mitochondria of tumor cells, Mito-ICT-4 downregulated SIRT3 protein expression, disrupted intracellular redox homeostasis, and led to a substantial increase in mitochondrial ROS levels, abnormal CypD-dependent MPTP opening, mitochondrial membrane potential depolarization, and ROS release into the cytoplasm, ultimately triggering ROS-mediated apoptosis in BEL-7402 cells. Transcriptomic analysis identified differentially expressed genes and enriched pathways, highlighting the ROS-mediated p38-MAPK signaling pathway as a key mediator of Mito-ICT-4-induced mitochondria-dependent apoptosis. The effects of Mito-ICT-4 on the expression of key genes (SIRT3, CypD, P-MKK6, P-P38, and DDIT3) were further validated by qRT-PCR and Western blot analysis, with results aligning with transcriptomic data. The novel ICT derivatives synthesized in this study, with mitochondria-targeting functionality, provide a basis for the development of targeted antitumor drugs.

## 1. Introduction

Cancer remains the second leading cause of death worldwide, representing a major global health challenge [1]. Mitochondria play a pivotal role in tumorigenesis by supporting tumor anabolism, regulating redox and calcium homeostasis, participating in transcriptional regulation, and modulating apoptosis, in addition to performing core biological functions [2,3]. Therefore, targeting tumor mitochondria is a promising approach for developing novel anticancer drugs with significant therapeutic potential.

Research has shown that the mitochondrial membrane potential of tumor cells is significantly higher than that of normal cells. According to the Nernst equation, for every 61.5 mV increase in Δ*ψ*m, the concentration of charged ions entering the inner membrane increases tenfold [4,5]. Electron-shifted lipophilic cationic compounds (DLCs) exploit the membrane potential difference between tumor and normal cells to selectively accumulate in tumor cell mitochondria. Consequently, DLC-conjugated small-molecule antitumor compounds are expected to specifically target tumor cells. Once inside the mitochondria, the released bioactive molecules enhance tumor cell selectivity and trigger apoptosis [6,7]. Currently, TPP^+^ is the most widely studied and applied DLC [8].

*Epimedium brevicornu* Maxim (*E. brevicornu*) is a traditional medicinal plant rich in isopentenyl flavonols, widely used in the treatment and prevention of various diseases. Icaritin (ICT) is derived from the hydrolysis of Icariin (ICA), the most abundant component in *E. brevicornu*. Its structure features an 8-isopentenyl flavonol backbone with three free phenolic hydroxyl groups at positions 3, 5, and 7 and exhibits a range of pharmacological activities [9]. Several studies have demonstrated the potent inhibitory effect of ICT, particularly against liver cancer [10,11,12]. Icaritin soft capsules were approved by the National Medical Products Administration (NMPA) of China in January 2022, primarily for the treatment of advanced hepatocellular carcinoma [13]. However, ICT is ineffective at low concentrations, and its reduced selectivity and cell permeability significantly limit its clinical application [14].

Therefore, we designed and synthesized a series of TPP^+^ derivatives by introducing alkyl chains of varying lengths onto the OH group at the C-7 position of ICT to enhance their antitumor activity and improve their selectivity for tumor cells while also initially investigating the antitumor mechanism of the optimal ICT derivative, Mito-ICT-4.

## 2. Results

### 2.1. Design and Synthesis

**Scheme 1:**
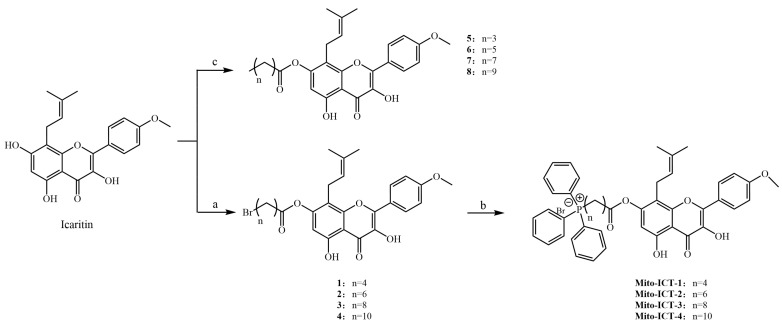
Synthetic route. Reagents and conditions: (**a**) EDCI, DMAP, 5-bromovaleric acid/7-bromoheptanoic acid/9-bromooctanoic acid/11-bromoundecanoic acid, DCM; (**b**) TPP, MeCN, 85 °C; (**c**) EDCI, DMAP, valeric acid/heptanoic acid/nonanoic acid/undecanoic acid, DCM.

Twelve novel ICT derivatives were designed and synthesized according to Scheme 1. In summary, ICT-TPP^+^ derivatives (Mito-ICT-1 to Mito-ICT-4) were synthesized through an esterification reaction between the 7-OH site of ICT and an alkyl chain containing a TPP^+^ group, with compounds **1**–**4** serving as intermediate products. To study the unique effects of the ICT-TPP^+^ derivatives, we also designed a series of fatty acid esters (**5**–**8**) bearing corresponding carbon chains for contrastive study. The structures of these new compounds were confirmed by ^1^H-NMR, ^13^C-NMR, and HRMS data. The spectral data are given below (Supplementary Figures S1–S12).

**1**: Pale yellow solid, yield: 36.6%. ^1^H NMR (300 MHz, Chloroform-*d*) δ11.72 (s, 1H, H-5), 8.17 (dd, *J* = 2.1 Hz, *J* = 6.9 Hz, 2H, H-2′, H-6′), 7.03 (dd, *J* = 2.1 Hz, *J* = 6.9 Hz, 2H, H-3′, H-5′), 6.53 (s, 1H, H-6), 5.14 (t, *J* = 6.6 Hz, 1H, H-12), 3.90 (s, 3H, 7′-OCH_3_), 3.49 (d, *J* = 4.5 Hz, 2H, H-11), 3.47 (m, 2H, H-5″), 2.66 (t, *J* = 7.5 Hz, 2H, H-2″), 1.97 (m, 2H, H-4″), 1.79 (m, 2H, H-3″), 1.69 (s, 6H, H-15, H-14). ^13^C NMR (75 MHz, Chloroform-*d*) δ175.54 (C-4), 170.47 (C-1″), 161.10 (C-4′), 157.87 (C-5), 153.58 (C-7), 153.35 (C-9), 146.42 (C-2), 135.79 (C-3), 132.44 (C-13), 129.33 (C-2′, C-6′), 122.85 (C-1′), 121.11 (C-12), 113.89 (C-3′, C-5′), 112.88 (C-8), 107.21 (C-10), 104.77 (C-6), 55.14 (C-7′), 33.93 (C-5″), 32.48 (C-2″), 31.57 (C-4″), 25.36 (C-14), 23.10 (C-3″), 22.41 (C-11), 17.85 (C-15). HRMS (ESI): *m/z* 531.1028 [M + H]^+^ (calculated for C_26_H_28_BrO_7_: 531.1018).

**2**: Pale yellow solid, yield: 43.5%. ^1^H NMR (300 MHz, Chloroform-*d*) δ11.72 (s, 1H, H-5), 8.17 (dd, *J* = 2.1 Hz, *J* = 6.9 Hz, 2H, H-2′, H-6′), 7.03 (dd, *J* = 2.1 Hz, *J* = 6.9 Hz, 2H, H-3′, H-5′), 6.53 (s, 1H, H-6), 5.15 (t, *J* = 6.6 Hz, 1H, H-12), 3.90 (s, 3H, 7′-OCH_3_), 3.45 (d, *J* = 4.5 Hz, 2H, H-11), 3.43 (m, 2H, H-7″), 2.62 (t, *J* = 7.5 Hz, 2H, H-2″), 1.90 (m, 2H, H-6″), 1.82 (m, 2H, H-3″), 1.69 (s, 6H, H-14, H-15), 1.53–1.46 (m, 4H, H-4″, H-5″). ^13^C NMR (75 MHz, Chloroform-*d*) δ175.95 (C-4), 171.34 (C-1″), 161.49 (C-4′), 158.25 (C-5), 154.10 (C-7), 153.71 (C-9), 146.83 (C-2), 134.85 (C-3), 132.75 (C-13), 128.96 (C-2′, C-6′), 123.27 (C-1′), 121.59 (C-12), 114.28 (C-3′, C-5′), 113.30 (C-8), 107.59 (C-10), 105.23 (C-6), 55.57 (C-7′), 34.17 (C-2″), 33.80 (C-7″), 32.61 (C-6″), 28.34 (C-4″), 27.92 (C-5″), 25.79 (C-14), 24.75 (C-3″), 22.83 (C-11), 18.26 (C-15). HRMS (ESI): *m/z* 559.1344 [M + H]^+^ (calculated for C_28_H_32_BrO_7_: 559.1331).

**3**: Pale yellow solid, yield: 51.0%. ^1^H NMR (300 MHz, Chloroform-*d*) δ11.71 (s, 1H, H-5), 8.17 (dd, *J* = 2.1 Hz, *J* = 6.9 Hz, 2H, H-2′, H-6′), 7.03 (dd, *J* = 2.1 Hz, *J* = 6.9 Hz, 2H, H-3′, H-5′), 6.53 (s, 1H, H-6), 5.16 (t, *J* = 6.6 Hz, 1H, H-12), 3.90 (s, 3H, 7′-OCH_3_), 3.45 (d, *J* = 4.5 Hz, 2H, H-11), 3.42 (m, 2H, H-9″), 2.60 (t, *J* = 7.5 Hz, 2H, H-2″), 1.87 (m, 2H, H-8″), 1.79 (m, 2H, H-3″), 1.69 (s, 6H, H-14, H-15), 1.46–1.38 (m, 8H, H-4″, H-5″, H-6″, H-7201D). ^13^C NMR (75 MHz, Chloroform-*d*) δ175.95 (C-4), 171.46 (C-1″), 161.48 (C-4′), 158.23 (C-5), 154.16 (C-7), 153.76 (C-9), 146.77 (C-2), 136.19 (C-3), 132.70 (C-13), 129.71 (C-2′, C-6′), 123.28 (C-1′), 121.64 (C-12), 114.28 (C-3′, C-5′), 113.31 (C-8), 107.55 (C-10), 105.26 (C-6), 55.52 (C-7′), 34.31 (C-2″), 34.05 (C-9″), 32.88 (C-8″), 29.20 (C-6″), 29.12 (C-5″), 28.70 (C-4″), 28.22 (C-7″), 25.77 (C-14), 24.92 (C-3″), 22.80 (C-11), 18.24 (C-15). HRMS (ESI): *m/z* 587.1652 [M + H]^+^ (calculated for C_30_H_36_BrO_7_: 587.1644).

**4**: Pale yellow solid, yield: 38.7%. ^1^H NMR (300 MHz, Chloroform-*d*) δ11.71 (s, 1H, H-5), 8.17 (dd, *J* = 2.1 Hz, *J* = 6.9 Hz, 2H, H-2′, H-6′), 7.03 (dd, *J* = 2.1 Hz, *J* = 6.9 Hz, 2H, H-3′, H-5′), 6.53 (s, 1H, H-6), 5.16 (t, *J* = 6.6 Hz, 1H, H-12), 3.90 (s, 3H, 7′-OCH_3_), 3.45 (d, *J* = 4.5 Hz, 2H, H-11), 3.41 (m, 2H, H-11″), 2.60 (t, *J* = 7.5 Hz, 2H, H-2″), 1.86 (m, 2H, H-10″), 1.75 (m, 2H, H-3″), 1.69 (s, 6H, H-14, H-15), 1.43–1.32 (m, 12H, H-4″, H-5″, H-6″, H-7″, H-8″, H-9″). ^13^C NMR (75 MHz, Chloroform-*d*) δ175.54 (C-4), 171.11 (C-1″), 161.07 (C-4′), 157.81 (C-5), 153.76 (C-7), 153.35 (C-9), 146.40 (C-2), 135.78 (C-3), 132.29 (C-13), 129.32 (C-2′, C-6′), 122.87 (C-1′), 121.20 (C-12), 113.87 (C-3′, C-5′), 112.91 (C-8), 107.19 (C-10), 104.86 (C-6), 55.15 (C-7′), 33.93 (C-2″), 33.74 (C-11″), 32.52 (C-10″), 29.07 (C-7″), 29.03 (C-5″), 28.92 (C-8″), 28.80 (C-4″), 28.45 (C-6″), 27.86 (C-9″), 25.36 (C-14), 24.55 (C-3″), 22.40 (C-11), 17.82 (C-15). HRMS (ESI): *m/z* 615.1942 [M + H]^+^ (calculated for C_32_H_40_BrO_7_: 615.1957).

**5**: Pale yellow solid, yield: 26.9%. ^1^H NMR (300 MHz, Chloroform-*d*) δ11.71 (s, 1H, H-5), 8.18 (dd, *J* = 2.1 Hz, *J* = 6.9 Hz, 2H, H-2′, H-6′), 7.04 (dd, *J* = 2.1 Hz, *J* = 6.9 Hz, 2H, H-3′, H-5′), 6.53 (s, 1H, H-6), 5.16 (t, *J* = 6.6 Hz, 1H, H-12), 3.90 (s, 3H, 7′-OCH_3_), 3.46 (d, *J* = 4.5 Hz, 2H, H-11), 2.61 (t, *J* = 7.5 Hz, 2H, H-2″), 1.79 (m, 2H, H-3″), 1.69 (s, 6H, H-14, H-15), 1.45 (m, 2H, H-4″), 0.99 (s, 3H, H-5″). ^13^C NMR (75 MHz, Chloroform-*d*) δ175.56 (C-4), 171.14 (C-1″), 161.07 (C-4′), 157.82 (C-5), 153.78 (C-7), 153.37 (C-9), 146.38 (C-2), 135.79 (C-3), 132.33 (C-13), 129.33 (C-2′, C-6′), 122.90 (C-1′), 121.19 (C-12), 113.88 (C-3′, C-5′), 112.94 (C-8), 107.15 (C-10), 104.84 (C-6), 55.14 (C-7′), 33.67 (C-2″), 26.62 (C-3″), 25.35 (C-15), 22.40 (C-4″), 21.98 (C-11), 17.81 (C-14), 13.44 (C-5″). HRMS (ESI): *m/z* 453.1931 [M + H]^+^ (calculated for C_26_H_29_O_7_: 453.1913).

**6**: Pale yellow solid, yield: 45.0%. ^1^H NMR (300 MHz, Chloroform-*d*) δ11.71 (s, 1H, H-5), 8.18 (dd, *J* = 2.1 Hz, *J* = 6.9 Hz, 2H, H-2′, H-6′), 7.04(dd, *J* = 2.1 Hz, *J* = 6.9 Hz, 2H, H-3′, H-5′), 6.53 (s, 1H, H-6), 5.16 (t, *J* = 6.6 Hz, 1H, H-12), 3.90 (s, 3H, 7′-OCH_3_), 3.47 (d, *J* = 4.5 Hz, 2H, H-11), 2.61 (t, *J* = 7.5 Hz, 2H, H-2″), 1.79 (m, 2H, H-3″), 1.69 (s, 6H, H-14, H-15), 1.43 (m, 2H, H-4″), 1.36–1.31 (m, 4H, H-5″, H-6″), 0.92 (s, 3H, H-7″). ^13^C NMR(75 MHz, Chloroform-*d*) δ176.00 (C-4), 171.56 (C-1″), 161.51 (C-4′), 158.26 (C-5), 154.23 (C-7), 153.81 (C-9), 146.81 (C-2), 136.23 (C-3), 132.73 (C-13), 129.76 (C-2′, C-6′), 123.35 (C-1′), 121.61 (C-12), 114.32 (C-3′, C-5′), 113.37 (C-8), 107.56 (C-10), 105.31 (C-6), 55.59 (C-7′), 34.39 (C-2″), 31.58 (C-5″), 28.94 (C-4″), 25.78 (C-14), 24.98 (C-3″), 22.84 (C-6″), 22.62 (C-11), 18.23 (C-15), 14.16 (C-7″). HRMS (ESI): *m/z* 481.2213 [M + H]^+^ (calculated for C_28_H_33_O_7_: 481.2226).

**7**: Pale yellow solid, yield: 46.9%. ^1^H NMR (300 MHz, Chloroform-*d*) δ11.71 (s, 1H, H-5), 8.18 (dd, *J* = 2.1 Hz, *J* = 6.9 Hz, 2H, H-2′, H-6′), 7.04 (dd, *J* = 2.1 Hz, *J* = 6.9 Hz, 2H, H-3′, H-5′), 6.54 (s, 1H, H-6), 5.16 (t, *J* = 6.6 Hz, 1H, H-12), 3.90 (s, 3H, 7′-OCH_3_), 3.48 (d, *J* = 4.5 Hz, 2H, H-11), 2.60 (t, *J* = 7.5 Hz, 2H, H-2″), 1.79 (m, 2H, H-3″), 1.69 (s, 6H, H-14, H-15), 1.40–1.30 (m, 10H, H-4″, H-5″, H-6″, H-7″, H-8″), 0.90 (s, 3H, H-9″). ^13^C NMR(75 MHz, Chloroform-*d*) δ175.99 (C-4), 171.66 (C-1″), 161.50 (C-4′), 158.26 (C-5), 154.22 (C-7), 153.76 (C-9), 146.89 (C-2), 136.22 (C-3), 132.72 (C-13), 129.76 (C-2′, C-6′), 123.33 (C-1′), 121.65 (C-12), 114.31 (C-3′, C-5′), 113.36 (C-8), 107.35 (C-10), 105.31 (C-6), 55.58 (C-7′), 34.39 (C-2″), 31.95 (C-7″), 29.85 (C-5″), 29.38 (C-6″), 29.27 (C-4″), 25.78 (C-14), 25.02 (C-3″), 22.79 (C-8″, C-11), 18.24 (C-15), 14.24 (C-9″). HRMS (ESI): *m/z* 509.2561 [M + H]^+^ (calculated for C_30_H_37_O_7_: 509.2539).

**8**: Pale yellow solid, yield: 42.3%. ^1^H NMR (300 MHz, Chloroform-*d*) δ11.71(s, 1H, H-5), 8.18 (dd, *J* = 2.1 Hz, *J* = 6.9 Hz, 2H, H-2′, H-6′), 7.04 (dd, *J* = 2.1 Hz, *J* = 6.9 Hz, 2H, H-3′, H-5′), 6.54 (s, 1H, H-6), 5.16 (t, *J* = 6.6 Hz, 1H, H-12), 3.90 (s, 3H, 7′-OCH_3_), 3.48 (d, *J* = 4.5 Hz, 2H, H-11), 2.60 (t, *J* = 7.5 Hz, 2H, H-2″), 1.78 (m, 2H, H-3″), 1.69 (s, 6H, H-14, H-15), 1.47 (m, 2H, H-10″), 1.43 (m, 2H, H-9″), 1.40–1.28 (m, 10H, H-4″, H-5″, H-6″, H-7″, H-8″), 0.89 (s, 3H, H-11″). ^13^C NMR (75 MHz, Chloroform-*d*) δ176.45 (C-4), 171.97 (C-1″), 161.93 (C-4′), 158.69 (C-5), 154.65 (C-7), 154.24 (C-9), 147.37 (C-2), 136.70 (C-3), 133.12 (C-13), 130.21 (C-2′, C-6′), 123.77 (C-1′), 122.08 (C-12), 114.71 (C-3′, C-5′), 113.76 (C-8), 108.13 (C-10), 105.72 (C-6), 55.96 (C-7′), 34.82 (C-2″), 32.46 (C-9″), 30.27 (C-7″), 30.13 (C-5″), 30.03 (C-8″), 29.86 (C-6″), 29.70 (C-4″), 26.19 (C-14), 25.45 (C-3″), 23.25 (C-11, C-10″), 18.66 (C-15), 14.67 (C-11″). HRMS (ESI): *m/z* 537.2841 [M + H]^+^ (calculated for C_32_H_41_O_7_: 537.2852).

**Mito-ICT-1**: Yellow sticky substance, yield: 48.7%. ^1^H NMR (300 MHz, Chloroform-*d*) δ11.70(s, 1H, H-5), 8.16 (dd, *J* = 2.1 Hz, *J* = 6.9 Hz, 2H, H-2′, H-6′), 7.89–7.77 (m, 9H, H-b, H-f, H-d), 7.72–7.66 (m, 6H, H-c, H-e), 7.03 (dd, *J* = 2.1 Hz, *J* = 6.9 Hz, 2H, H-3′, H-5′), 6.27 (s, 1H, H-6), 5.06 (t, *J* = 6.6 Hz, 1H, H-12), 3.89 (s, 3H, 7′-OCH_3_), 3.85 (m, 2H, H-5″), 3.40 (d, *J* = 4.5 Hz, 2H, H-11), 2.74 (t, *J* = 7.5 Hz, 2H, H-2″), 1.80 (m, 2H, H-3″), 1.73 (s, 3H, H-14), 1.71 (s, 3H, H-15), 1.61 (m, 2H, H-4″). ^13^C NMR (75 MHz, Chloroform-*d*) δ175.43 (C-4), 170.65 (C-1″), 161.04 (C-4′), 157.55 (C-5), 153.34 (C-7), 153.17 (C-9), 146.42 (C-2), 135.71 (C-3), 134.77 (C-d), 133.34 (C-b, C-f), 130.27 (C-c, C-e), 129.22 (C-2′, C-6′), 122.69 (C-13), 120.82 (C-a), 118.39 (C-12), 117.26 (C-1′), 113.80 (C-3′, C-5′), 112.81 (C-8), 107.04 (C-10), 104.50 (C-6), 55.09 (C-7′), 32.73 (C-2″), 25.24 (C-14), 25.00 (C-4″), 24.79 (C-11), 22.24 (C-3″), 21.46 (C-5″), 17.76 (C-15). HRMS (ESI): *m/z* 713.2682 [M − Br]^+^ (calculated for C_44_H_42_O_7_P^+^: 713.2663).

**Mito-ICT-2**: Yellow sticky substance, yield: 50.4%. ^1^H NMR (300 MHz, Chloroform-*d*) δ11.70 (s, 1H, H-5), 8.16 (dd, *J* = 2.1 Hz, *J* = 6.9 Hz, 2H, H-2′, H-6′), 7.88–7.77 (m, 9H, H-b, H-f, H-d), 7.72–7.66 (m, 6H, H-c, H-e), 7.03 (dd, *J* = 2.1 Hz, *J* = 6.9 Hz, 2H, H-3′, H-5′), 6.46 (s, 1H, H-6), 5.10 (t, *J* = 6.6 Hz, 1H, H-12), 3.89 (s, 3H, 7′-OCH_3_), 3.84 (m, 2H, H-7″), 3.44 (d, *J* = 4.5 Hz, 2H, H-11), 2.58 (t, *J* = 7.5 Hz, 2H, H-2″), 1.80 (m, 2H, H-3″), 1.75 (s, 6H, H-14, H-15), 1.69 (m, 2H, H-6″), 1.64 (m, 2H, H-5″), 1.46 (m, 2H, H-4″). ^13^C NMR (75 MHz, Chloroform-*d*) δ175.41 (C-4), 170.98 (C-1″), 160.99 (C-4′), 157.55 (C-5), 153.53 (C-7), 153.18 (C-9), 146.35 (C-2), 135.67 (C-3), 134.68 (C-d), 133.31 (C-b, C-f), 130.22 (C-c, C-e), 129.20 (C-2′, C-6′), 122.69 (C-13), 120.94 (C-a), 118.46 (C-12), 117.32 (C-1′), 113.78 (C-3′, C-5′), 112.85 (C-8), 106.99 (C-10), 104.66 (C-6), 55.10 (C-7′), 33.50 (C-2″), 29.64 (C-4″), 29.42 (C-6″), 29.29 (C-5″), 25.27 (C-14), 23.90 (C-3″), 22.50 (C-11), 22.26 (C-7″), 17.77 (C-15). HRMS (ESI): *m/z* 741.2988 [M − Br]^+^ (calculated for C_46_H_46_O_7_P^+^: 741.2976).

**Mito-ICT-3**: Yellow– sticky substance, yield: 42.4%. ^1^H NMR (300 MHz, Chloroform-*d*) δ11.70 (s, 1H, H-5), 8.16 (dd, *J* = 2.1 Hz, *J* = 6.9 Hz, 2H, H-2′, H-6′), 7.87–7.76 (m, 9H, H-b, H-f, H-d), 7.72–7.66 (m, 6H, H-c, H-e), 7.03 (dd, *J* = 2.1 Hz, *J* = 6.9 Hz, 2H, H-3′, H-5′), 6.49 (s, 1H, H-6), 5.13 (t, *J* = 6.6 Hz, 1H, H-12), 3.89 (s, 3H, 7′-OCH_3_), 3.86 (m, 2H, H-9″), 3.46 (d, *J* = 4.5 Hz, 2H, H-11), 2.56 (t, *J* = 7.5 Hz, 2H, H-2″), 1.76 (s, 6H, H-14, H-15), 1.71 (m, 2H, H-3″), 1.66 (m, 2H, H-8″), 1.47–1.30 (m, 8H, H-4″, H-5″, H-6″, H-7″). ^13^C NMR (75 MHz, Chloroform-*d*) δ175.47 (C-4), 171.07 (C-1″), 161.05 (C-4′), 157.63 (C-5), 153.67 (C-7), 153.22 (C-9), 146.40 (C-2), 135.70 (C-3), 134.69 (C-d), 133.33 (C-b, C-f), 130.24 (C-c, C-e), 129.26 (C-2′, C-6′), 122.76 (C-13), 121.02 (C-a), 118.57 (C-12), 117.43 (C-1′), 113.84 (C-3′, C-5′), 112.91 (C-8), 107.04 (C-10), 104.68 (C-6), 55.13 (C-7′), 33.74 (C-2″), 30.04 (C-5″), 29.83 (C-4″), 29.32 (C-8″), 28.49 (C-6″), 25.29 (C-14), 24.33 (C-3″), 22.59 (C-7″), 22.29 (C-11), 21.92 (C-9″), 17.77 (C-15). HRMS (ESI): *m/z* 769.3274 [M − Br]^+^ (calculated for C_48_H_50_O_7_P^+^: 769.3289).

**Mito-ICT-4**: Yellow sticky substance, yield: 49.7%. ^1^H NMR (300 MHz, Chloroform-*d*) δ11.70 (s, 1H, H-5), 8.16 (dd, *J* = 2.1 Hz, *J* = 6.9 Hz, 2H, H-2′, H-6′), 7.87–7.76 (m, 9H, H-b, H-f, H-d), 7.72–7.66 (m, 6H, H-c, H-e), 7.03 (dd, *J* = 2.1 Hz, *J* = 6.9 Hz, 2H, H-3′, H-5′), 6.50 (s, 1H, H-6), 5.13 (t, *J* = 6.6 Hz, 1H, H-12), 3.89 (s, 3H, 7′-OCH_3_), 3.75 (m, 2H, H-11″), 3.46 (d, *J* = 4.5 Hz, 2H, H-11), 2.57 (t, *J* = 7.5 Hz, 2H, H-2″), 1.77 (s, 6H, H-14, H-15), 1.72 (m, 2H, H-3″), 1.66 (m, 2H, H-10″), 1.47–1.36 (m, 12H, H-4″, H-5″, H-6″, H-7″, H-8″, H-9″). ^13^C NMR (75 MHz, Chloroform-*d*) δ175.84 (C-4), 171.47 (C-1″), 161.39 (C-4′), 158.01 (C-5), 154.03(C-7), 153.70 (C-9), 146.52 (C-2), 136.12 (C-3), 135.09 (C-d), 133.68 (C-b, C-f), 130.63 (C-c, C-e), 129.60 (C-2′, C-6′), 123.10 (C-13), 121.35 (C-a), 118.87 (C-12), 117.74 (C-1′), 114.18 (C-3′, C-5′), 113.26 (C-8), 107.40 (C-10), 105.05 (C-6), 55.46 (C-7′), 34.18 (C-2″), 30.55 (C-6″), 30.34 (C-5″), 29.69 (C-7″), 29.24 (C-4″), 29.12 (C-10″), 29.00 (C-8″), 25.66 (C-14), 24.80 (C-3″), 23.00 (C-9″), 22.67 (C-11), 22.33 (C-11″), 18.13 (C-15). HRMS (ESI): *m/z* 797.3615 [M − Br]^+^ (calculated for C_50_H_54_O_7_P^+^: 797.3602).

### 2.2. In Vitro Biological Activity

We assessed the in vitro antitumor activity of all derivatives in four tumor cell lines (A549, PC-3M, MCF-7, and BEL-7402) and one normal cell line (HEK293) using the MTT assay, employing 5-fluorouracil (5-FU) and doxorubicin (DOX) as positive controls. The results indicated (Table 1) that the antitumor activities of the intermediates (derivatives **1**–**4**) and fatty acid ester derivatives (derivatives **5**–**8**) were generally weak, and many of the derivatives failed to achieve an effective concentration (i.e., IC_50_ value < 100 μmol/L) in some tumor cell lines. Among the three tumor cell lines (A549, PC-3M, and MCF-7), derivatives **1**–**3** demonstrated antitumor activity comparable to ICT. However, all ICT-TPP^+^ derivatives exhibited significantly enhanced antitumor activity relative to ICT across the four tumor cell lines. Nearly half of the ICT-TPP^+^ derivatives exhibited greater potency than the two positive control drugs. In particular, in the BEL-7402 cell line, Mito-ICT-4 exhibited an IC_50_ value of 0.73 ± 0.06 μM, approximately 29 times lower than that of ICT, demonstrating exceptionally potent antitumor activity. In contrast, the ICT-TPP^+^ derivatives displayed lower cytotoxicity than ICT in HEK293 cells, with the IC_50_ value of Mito-ICT-4 in HEK293 cells measured at 67.11 ± 2.09 μM, representing a 55.4% reduction compared to ICT. Based on these findings, Mito-ICT-4 and BEL-7402 cells were chosen for further investigation.

### 2.3. Aggregation and Distribution of Mito-ICT-4 in Mitochondria of BEL-7402 Cells

**Figure 1:**
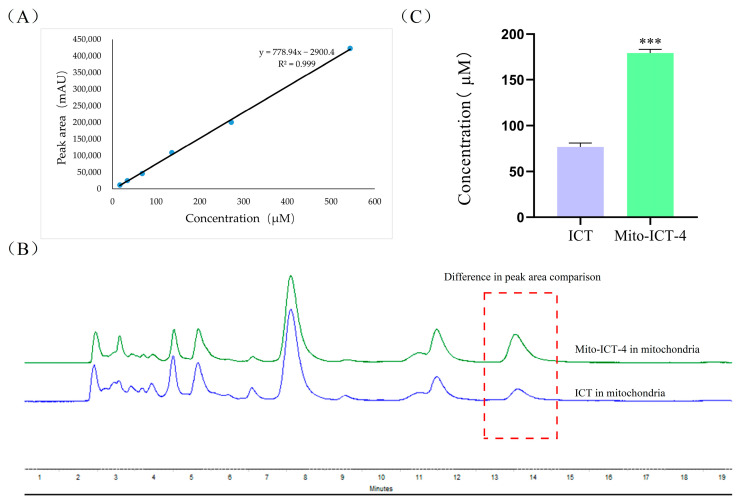
Aggregation and distribution of compounds ICT and Mito-ICT-4 in mitochondria of BEL-7402 cells. (**A**) Concentration-peak area curves of the standards of ICT; (**B**) Liquid chromatogram of the mitochondrial extracts of BEL-7402 cells after treatment of ICT and Mito-ICT-4 standards with 10 μM ICT and Mito-ICT-4 for 7 h; (**C**) Histogram of the concentration of the drugs in the mitochondria. The values are means ± SD from three independent experiments. *** *p* < 0.001 vs. control.

To further compare the mitochondrial targeting capacities of Mito-ICT-4 and ICT in tumor cells, the differential content within the mitochondria of BEL-7402 cells was quantified using HPLC. The results (Figure 1) demonstrated that the Mito-ICT-4 administration group exhibited antitumor activity through hydrolysis to ICT upon entering the mitochondria. Moreover, the aggregation of Mito-ICT-4 in the mitochondria was approximately 2.5 times higher than that of ICT (*p* < 0.001), which aligned with the trial design expectations.

### 2.4. Effect of Mito-ICT-4 on Mitochondrial Membrane Potential in BEL-7402 Cells

**Figure 2:**
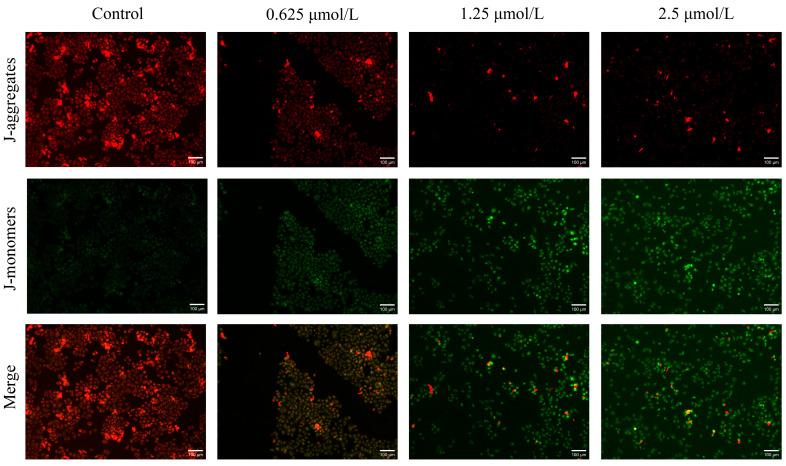
The effect of Mito-ICT-4 on the mitochondrial membrane potential of BEL-7402 cells. The values are means ± SD from three independent experiments. Colocalization of Mito-ICT-4 with mito-tracker red in BEL-7402 cells was imaged by confocal microscopy. Scale bar: 100 μm.

The results (Figure 2) indicated that the cell membrane potential of the control group was relatively high, leading to the formation of polymers and the generation of red fluorescence. In contrast, compared to the control group, most cells in the administration group were monomers exhibiting green fluorescence, with fluorescence intensity increasing with concentration. This suggests that Mito-ICT-4 led to a reduction in the mitochondrial membrane potential of cells, resulting in damage to the mitochondria of BEL-7402 cells.

### 2.5. Mito-ICT-4 Induces Apoptosis in BEL-7402 Cells

**Figure 3:**
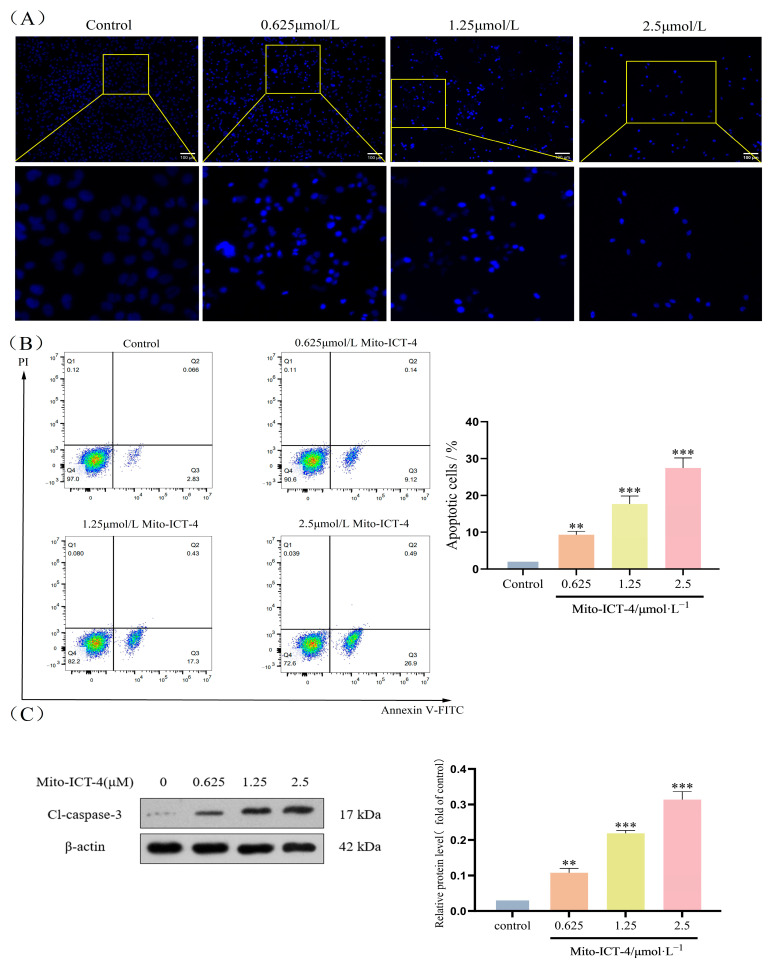
The effect of Mito-ICT-4 on apoptosis of BEL-7402 cells. (**A**) DAPI staining method to observe apoptosis, the nuclei indicated by arrows showed the phenomenon of crumpling and cleavage, and the fluorescence was enhanced; (**B**) Schematic diagram of apoptosis obtained by flow cytometry analysis; (**C**) Effect of Mito-ICT-4 on the level of Cl-caspase-3. The values are means ± SD from three independent experiments. ** *p* < 0.01 vs. control, *** *p* < 0.001 vs. control.

The results showed (Figure 3A) that the cells in the control group exhibited a uniform morphological structure with low fluorescence intensity. After 48 h of treatment, the cells began to undergo apoptosis. As the drug concentration increased, a significant reduction in the number of cells was observed under fluorescence microscopy, and their morphology displayed incomplete characteristics. In addition, the nuclei exhibited crumpling and cleavage, along with a significant increase in fluorescence intensity, a phenomenon indicating changes in the morphological characteristics of the nuclei during apoptosis. The Annexin V-FITC/PI flow cytometry assay (Figure 3B) revealed that all concentrations of Mito-ICT-4 significantly promoted apoptosis compared to the control group, particularly the total apoptosis rate of 27.39% in the high-dose group. The results of Western blot experiments (Figure 3C, Supplementary Figure S13) indicated that the expression of Cl-caspase-3 was elevated in the low-concentration group compared to the control group (*p* < 0.01), while its expression was significantly higher in the high-concentration group in the Mito-ICT-4 treated cells (*p* < 0.001).

### 2.6. Molecular Docking Result

**Figure 4:**
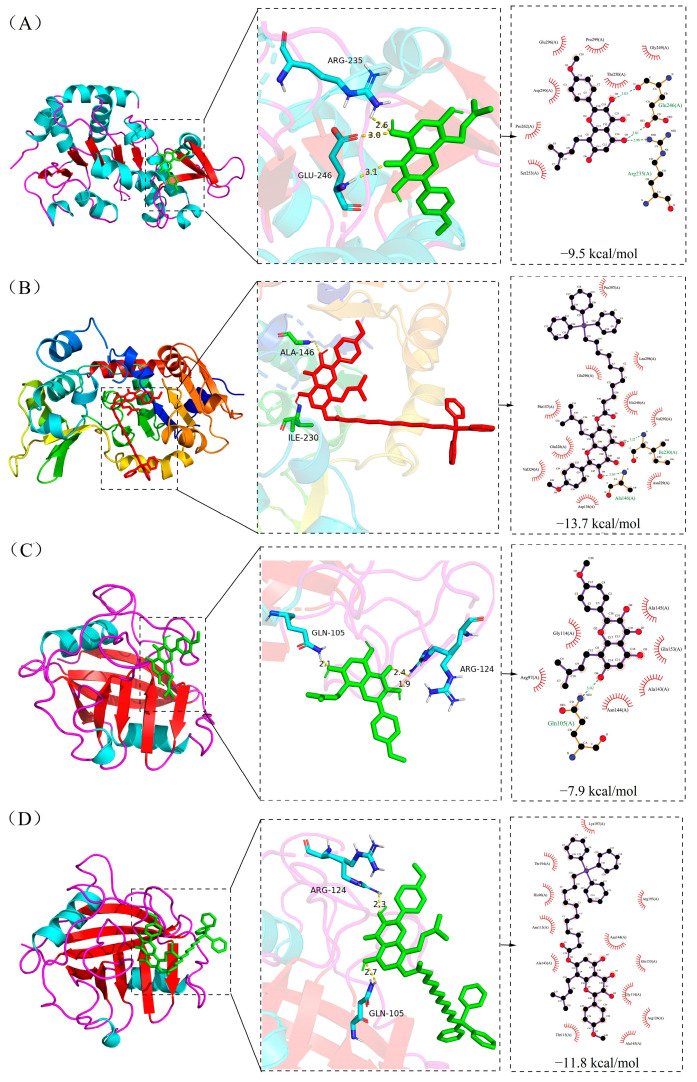
Molecular docking results. (**A**) Docking result plots of ICT with SIRT3; (**B**) Docking result plots of Mito-ICT-4 with SIRT3; (**C**) Docking result plots of ICT with CypD; (**D**) Docking result plots of Mito-ICT-4 with CypD.

Molecular docking technology was employed to simulate and predict the mechanism of action of Mito-ICT-4 on BEL-7402 cells. As shown in Table 2, the binding energy of Mito-ICT-4 to MKK6, P38, and DDIT3 was higher than that of ICT, whereas its binding energy to SIRT3 and CypD was lower. Additionally, the docking interactions of SIRT3 and CypD were visualized, as presented in Figure 4.

### 2.7. Effect of Mito-ICT-4 on Intracellular ROS in BEL-7402 Cells

**Figure 5:**
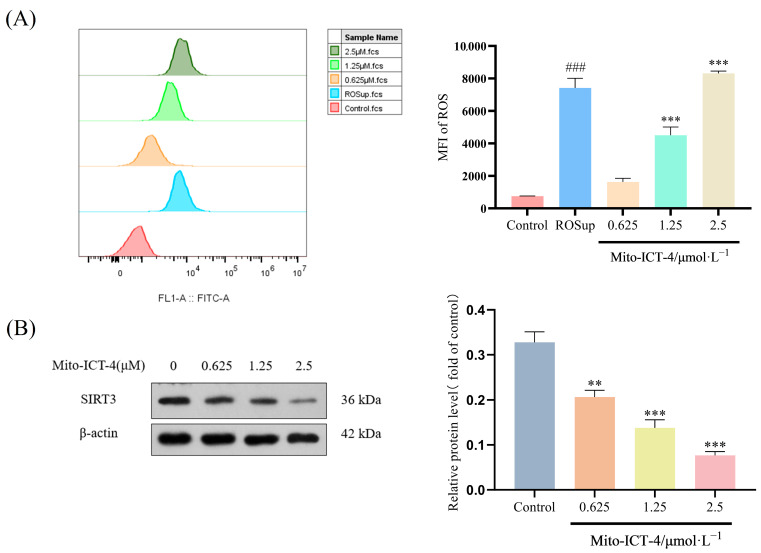
The effect of Mito-ICT-4 on intracellular ROS levels in BEL-7402 cells. (**A**) Flow cytometry to detect the effect of Mito-ICT-4 on ROS content in BEL-7402 cells, ROSup was the positive control group; (**B**) Effect of Mito-ICT-4 on the expression of SIRT3 protein level. The values are means ± SD from three independent experiments. ^###^ *p* < 0.01 vs. control, ** *p* < 0.01 vs. control, *** *p* < 0.001 vs. control.

The effect of Mito-ICT-4 on reactive oxygen species (ROS) levels in BEL-7402 cells was assessed using flow cytometry, with ROSup serving as a positive control. Following the 48-h treatment of the cells with Mito-ICT-4, as shown in (Figure 5A), the intracellular ROS levels in BEL-7402 cells were significantly elevated, and the ROS levels exhibited a dose-dependent increase with the rising drug concentration. Western blot (Figure 5B, Supplementary Figure S14) analysis of SIRT3, a critical protein involved in ROS regulation within mitochondria, showed that in Mito-ICT-4-treated cells, SIRT3 expression levels were decreased in the low-concentration group (*p* < 0.01), and significantly reduced in the medium- and high-concentration groups, relative to the control group (*p* < 0.001). Consistent with the molecular docking results, this suggests that Mito-ICT-4 may trigger changes in ROS levels by regulating SIRT3.

### 2.8. Effect of Mito-ICT-4 on Mitochondrial Membrane Permeability in BEL-7402 Cells

**Figure 6:**
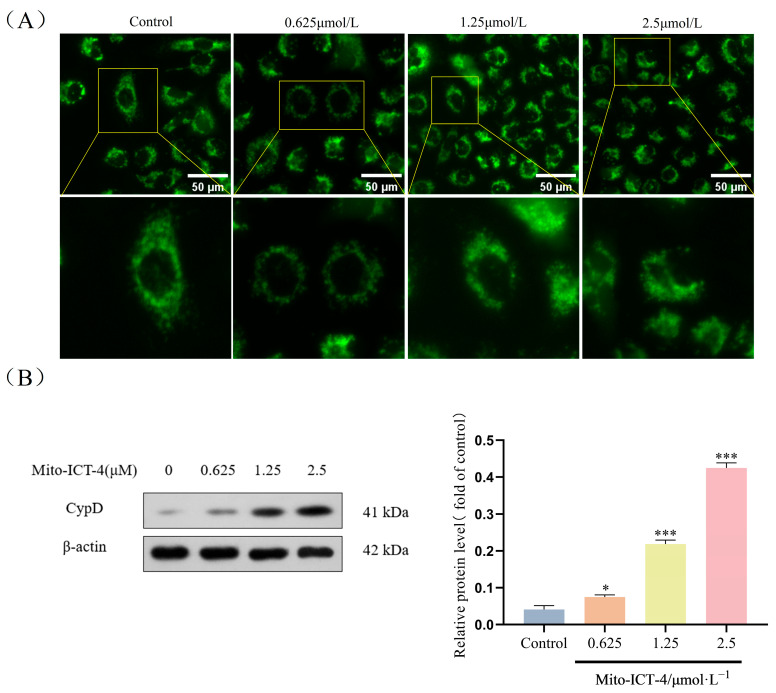
The effect of Mito-ICT-4 on mitochondrial membrane permeability in BEL-7402 cells. (**A**) Measurement of mitochondrial permeability transition pore opening with Calcein AM and CoCl_2_; (**B**) Effect of Mito-ICT-4 on CypD protein level expression. The values are means ± SD from three independent experiments. * *p* < 0.05 vs. control, *** *p* < 0.001 vs. control.

As illustrated in the experimental results (Figure 6A), the control group exhibited a distinct oval shape. As the drug concentration increased, the morphology progressively transitioned from oval to round and, ultimately, to an irregular shape. CypD is a critical component of the MPTP protein complex. Western blot results (Figure 6B, Supplementary Figure S15) demonstrated that, in Mito-ICT-4-treated cells, CypD protein expression was elevated in the low-concentration group compared to the control group (*p* < 0.01), while CypD expression was significantly higher in the medium and high-concentration groups (*p* < 0.001). CypD is a critical component of the MPTP protein complex. These findings align with the molecular docking results, suggesting that Mito-ICT-4 may elevate CypD protein levels, leading to abnormal MPTP opening, increased membrane permeability, and progressive loss of mitochondrial structural integrity.

### 2.9. Transcriptome Sequencing Results

**Figure 7:**
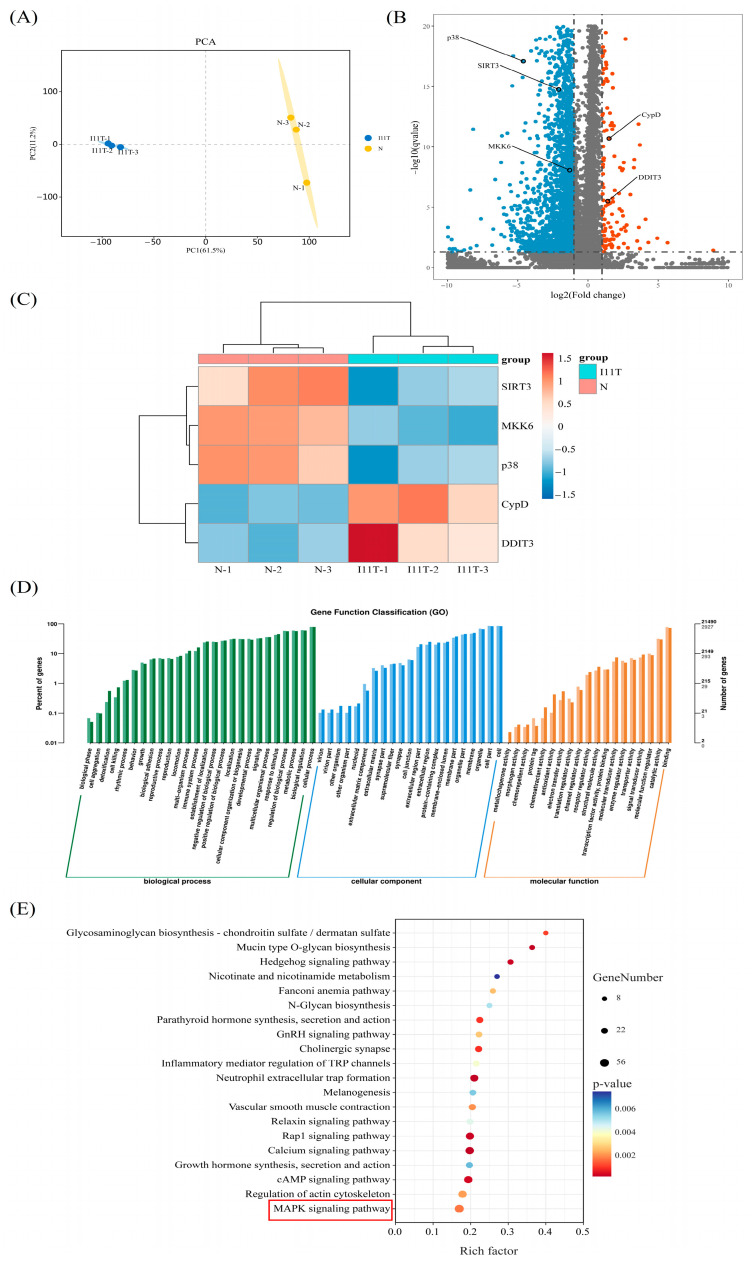
Transcriptome sequencing analysis. (**A**) Principal component (PCA) analysis plot; (**B**) volcano plot of differentially expressed genes; (**C**) Heatmap of key differential expression analyzed by RNAseq. (**D**) GO analysis; (**E**) KEGG pathway analysis. The experimental results were repeated more than three times. The values are means ± SD from three independent experiments.

PCA demonstrated clear separation between samples from different groups and exhibited high biological reproducibility within the same group (Figure 7A). The volcano plot results indicated that Mito-ICT-4 treatment led to the upregulation of 197 genes and the downregulation of 2887 genes in BEL-7402 cells compared to the control group (Figure 7B). Heatmaps following the clustering of key differential genes clearly illustrate the role of Mito-ICT-4 in gene expression regulation (Figure 7C). GO analysis revealed functional enrichment of differentially expressed genes across biological processes, cellular components, and molecular functions. In the top-10 GO in terms of differential gene enrichment, biological processes, including cellular processes, bioregulation, and metabolic processes, were significantly influenced by Mito-ICT-4. Alterations in cellular components were primarily associated with membrane fractions, membrane lumen, and protein-containing complexes. Furthermore, significant differences in molecular functions, such as catalytic activity, electron transport activity, and channel regulator activity, were identified (Figure 7D). KEGG pathway enrichment analysis of the 3084 differentially expressed genes identified key pathways associated with these genes, with these genes primarily enriched in the MAPK signaling pathway, calcium signaling pathway, and cAMP signaling pathway, suggesting that these pathways may play crucial roles in mediating the antitumor activity of Mito-ICT-4 (Figure 7E).

### 2.10. qRT-PCR Validation of Key Differential Genes

**Figure 8:**
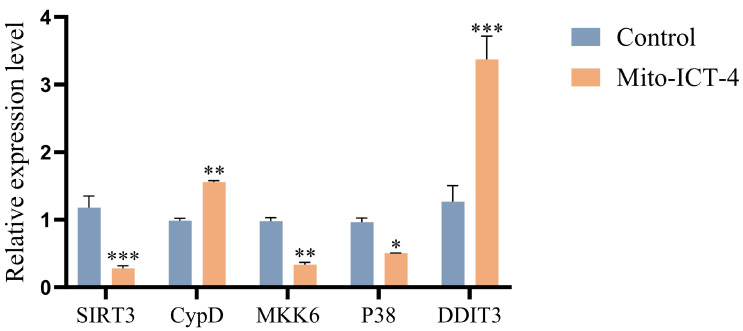
The effect of Mito-ICT-4 on the transcription of key differential genes in BEL-7402 cells. The values are means ± SD from three independent experiments. * *p* < 0.05 vs. control, ** *p* < 0.01 vs. control, *** *p* < 0.001 vs. control.

In order to verify the transcriptome sequencing results and further investigate whether the MAPK signaling pathway is involved in Mito-ICT-4-induced apoptosis in BEL-7402 cells, key candidate genes in the MAPK signaling pathway were selected from among 3084 differentially expressed genes. Subsequently, qRT-PCR was performed on the differentially expressed genes *SIRT3*, *CypD*, *MKK6*, *P38*, and *DDIT3*, using *β-actin* as the internal control. The results (Figure 8, Supplementary Table S1) showed that, compared with the blank control group, the expression levels of *SIRT3*, *MKK6*, and *P38* were significantly downregulated, whereas those of *CypD* and *DDIT3* were significantly upregulated. These results were consistent with the transcriptome data and demonstrated statistical significance (*p* < 0.05).

### 2.11. Western Blot Result

**Figure 9:**
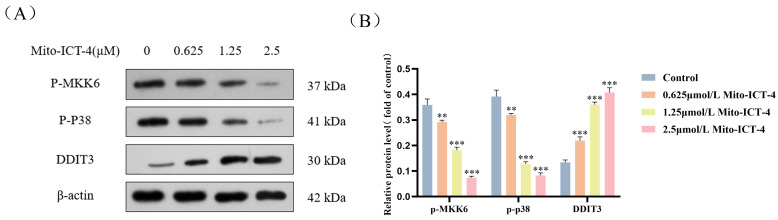
Western blot analysis of the effect of Mito-ICT-4 on MAPK pathway-related proteins. (**A**) Effect of Mito-ICT-4 on the expression levels of MAPK signaling pathway-related proteins. (**B**) Statistical analysis of protein expression levels. The values are means ± SD from three independent experiments. ** *p* < 0.01 vs. control, *** *p* < 0.001 vs. control.

Based on the transcriptome sequencing and qRT-PCR results, the protein expression levels of P-MKK6, P-P38, and DDIT3 were further validated using Western blot analysis, with β-actin as the internal control. The results (Figure 9, Supplementary Figure S16–S19) showed that, compared with the blank control group, the expression levels of P-P38 and P-MKK6 in Mito-ICT-4-treated BEL-7402 cells decreased significantly with increasing concentrations (*p* < 0.001), whereas the expression level of DDIT3 increased significantly (*p* < 0.001). These findings were consistent with the transcriptome and qRT-PCR results. These results further demonstrate that Mito-ICT-4 induces apoptosis in BEL-7402 cells by modulating the phosphorylation of proteins in the p38 MAPK signaling pathway in a concentration-dependent manner.

## 3. Discussion

Liver cancer ranks as the sixth most common cancer worldwide and has the second highest tumor-related mortality. The survival rate for liver cancer is exceedingly low due to challenges in early diagnosis, its rapid progression, and the lack of effective targeted therapies. Therefore, the search for safe and effective targeted drugs is currently a hot spot in clinical research [15].

This study focused on the design and synthesis of twelve novel ICT derivatives. Given the presence of free phenolic hydroxyl groups at the C-3, C-5, and C-7 positions in the ICT chemical structure and the established higher reactivity of the 7-OH group compared to the 3-OH and 5-OH groups, as well as evidence from the literature [16,17] indicating the preference for this position in anticancer chemical modifications, the 7-OH position was selected for esterification with a TPP^+^ alkyl chain. This approach successfully resulted in the preparation of ICT-TPP^+^ derivatives, with fatty acid derivatives synthesized concurrently as a control. In the synthesis of intermediates and fatty acid ester derivatives, it was observed that when 1.5–2.0 equivalents of bromic acid and fatty acids were used, the reaction exhibited low selectivity between the 3-, 5-, and 7-OH groups, resulting in a mixture of mono- and disubstituted products. Above 3.0 equivalents, only 3,7-OH disubstituted products were produced. The optimum feeding ratio of 1:1.2 was determined based on the yield and separation difficulty, at which point the 7-OH monosubstituted derivatives exhibited the highest selectivity [17]. For the synthesis of ICT-TPP^+^ derivatives, based on the conditions described in the literature [18], when the feeding ratio of bromate derivatives to triphenylphosphine was 1:5, the reaction was conducted at 80 °C in acetonitrile, with reflux for 48 h. It was observed that the reaction was accompanied by continuous cleavage of the ester bond in the bromate derivatives and that residual TPP^+^ feedstock could not be fully removed in the subsequent separation process. To optimize the reaction, the reaction conditions were modified in this study, and the optimal conditions were identified: the feeding ratio of bromate derivatives to TPP^+^ was 1:3.5, the reaction solvent was anhydrous acetonitrile, the reaction temperature was 85 °C, the reaction time was 60 h, and the yields ranged from 42.4% to 50.4%, which were higher than those reported in the literature. The optimized process not only increased the yield of the target product but also minimized the formation of by-products, providing a reliable foundation for subsequent studies.

Activity screening revealed that all ICT-TPP^+^; derivatives exhibited varying degrees of enhanced antitumor activity compared to ICT across four tumor cell lines, with notably higher selectivity toward BEL-7402 cells. These results underscore the significance of incorporating the TPP^+^; moiety to improve the biological activity of ICT. The conformational relationship suggests that the length of the alkyl carbon chain linking ICT and TPP^+^; plays a crucial role in determining antitumor potency. Overall, longer alkyl carbon chains tended to confer stronger antitumor activity, potentially due to spatial constraints limiting the efficacy of shorter chains [19]. However, the same ICT-TPP^+^ derivatives exhibited variable IC_50_ values across different tumor cell lines. Analysis revealed that the observed antitumor efficacy correlated with the intrinsic IC_50_ value of ICT in each specific cell line. Furthermore, the ICT-TPP^+^; derivatives were markedly more toxic to tumor cells than to normal cells and showed reduced cytotoxicity relative to ICT, indicating a degree of selectivity between tumor and normal cells. This result is consistent with the findings of Song et al. [18], who mitigated the toxicity of triptolide by chemically introducing a TPP^+^; group into its structure. In particular, Mito-ICT-4 exhibited approximately 33-fold selectivity for tumor cells, with an IC_50_ of 0.73 ± 0.06 μM in BEL-7402 cells compared to 67.11 ± 2.09 μM in HEK293 cells.

This study investigated the detailed mechanism of action of Mito-ICT-4. Using an HPLC method to detect mitochondrial aggregation, we observed that the accumulation of Mito-ICT-4 in the mitochondria of BEL-7402 cells was approximately 2.5 times greater than that of ICT. This result aligns with the findings of Ma et al. [20], who reported a 2.33–2.90-fold increase in mitochondrial targeting capability in their study of ginsenoside-TPP^+^ conjugates. These findings suggest that TPP^+^ effectively facilitates the delivery of ICT to the mitochondria of tumor cells, thereby enhancing its mitochondrial targeting. Further studies revealed that Mito-ICT-4 was hydrolyzed to ICT after entering the mitochondria of BEL-7402 cells, likely due to the weakly alkaline nature of the mitochondrial matrix (pH ≈ 8.0), and the high enzymatic activity of enzymes (e.g., esterase, phosphatase) in certain regions, which hydrolyze specific chemical bonds (e.g., ester and phosphate bonds) [21,22].

In the investigation of apoptosis mechanisms, BEL-7402 cells treated with Mito-ICT-4 displayed incomplete bright blue fluorescence upon DAPI staining, with 27.39% of the cells undergoing early apoptosis. The expression level of the apoptotic biomarker CL-caspase-3 protein was upregulated. These results indicate that Mito-ICT-4 may inhibit the proliferation of BEL-7402 cells by inducing apoptosis. This is similar to the mechanism reported by Li et al. [23], where ICT induces apoptosis in liver cancer cells by activating the Caspase cascade. However, this study further reveals the enhanced effect brought by the introduction of TPP^+^, showing that the apoptosis-inducing ability of Mito-ICT-4 is approximately 29.8 times greater than that of ICT [24].

ROS plays a crucial role in the initiation and execution of apoptosis in cancer cells [25,26]. SIRT3, a major mitochondrial deacetylase, plays a pivotal role in regulating ROS levels and mitigating oxidative damage to cellular components; it is also an emerging target for antitumor drug development [27]. Our results demonstrate that after Mito-ICT-4 enters the mitochondria of BEL-7402 cells, it downregulates SIRT3 levels, disrupting the intracellular redox balance and increasing ROS levels. This finding is consistent with the results of Zeng et al. [28], who reported elevated mitochondrial ROS levels in SIRT3-deficient mice. However, this study unveils a novel mechanism by which ICT exerts its anticancer effect through the regulation of the SIRT3–ROS axis. CypD acts as a key regulator of MPTP opening, and its associated opening plays a crucial role in ROS-induced mitochondrial dysfunction and apoptosis [29]. When ROS levels are abnormally elevated in mitochondria, CypD protein expression increases, resulting in abnormal MPTP opening, which releases mitochondrial ROS into the cytoplasm and activates ROS-mediated apoptotic signaling [30,31]. In our study, we found that after Mito-ICT-4 enters the mitochondria of BEL-7402 cells, it significantly promotes CypD expression, further triggering abnormal MPTP opening, disrupting mitochondrial structural integrity, releasing ROS, and decreasing mitochondrial membrane potential, ultimately triggering cell apoptosis. This is similar to the mechanism reported by Hou et al. [32], where the cationic antimicrobial peptide NRC-03 induces apoptosis in oral squamous cell carcinoma cells through the CypD-MPTP axis-mediated mitochondrial oxidative stress.

Numerous studies have shown that ROS are closely associated with the mitogen-activated protein kinase (MAPK) signaling pathway. The accumulation of ROS may induce apoptosis by activating the MAPK signaling cascade. Within this pathway, c-Jun N-terminal kinase (JNK), extracellular signal-regulated kinase 1/2 (Erk1/2), and p38 MAPK are sequentially activated through phosphorylation, with the p38 MAPK signaling pathway being the primary regulator of cell growth and apoptosis [33,34]. For example, ICT can induce cell apoptosis in breast cancer, endometrial cancer, and lung cancer by regulating the MAPK signaling pathways, specifically through the sustained phosphorylation of ERK and p38, without affecting the phosphorylation of JNK. This regulation leads to the modulation of cyclin D1, Bax, and Bcl expression, promoting the cleavage of Caspase-3 and PARP [35]. To further elucidate the molecular mechanism of ROS-mediated Mito-ICT-4-induced apoptosis, we performed transcriptome sequencing analysis to assess the impact of Mito-ICT-4 on the gene expression profile in BEL-7402 cells. Sequencing analysis revealed that, after 48 h of Mito-ICT-4 treatment, 197 genes were upregulated, and 2887 genes were downregulated. KEGG enrichment analysis of the differentially expressed genes indicated significant enrichment in the MAPK signaling pathway, which ranked first in terms of significance. Based on the literature and the results of this study, it is hypothesized that Mito-ICT-4 influences apoptosis in BEL-7402 cells by regulating the MAPK signaling pathway. This finding is similar to the results of Rahmani et al. [36], which showed that resveratrol activates the MAPK signaling pathway (including JNK, p38, and ERK) by increasing intracellular ROS levels, ultimately inducing apoptosis in BEL-7402 cells.

To validate the results of transcriptome sequencing and investigate the potential involvement of the MAPK signaling pathway in Mito-ICT-4-induced apoptosis of BEL-7402 cells, we selected key candidate genes related to the MAPK pathway (*SIRT3*, *CypD*, *MKK6*, *P38*, and *DDIT3*) from the differentially expressed gene set. Molecular docking was employed to conduct molecular simulations, which revealed that the binding affinity of Mito-ICT-4 to MKK6, P38, and DDIT3 was potentially weaker than that of ICT, whereas its binding to SIRT3 and CypD was stronger. These findings suggest that MKK6, P38, and DDIT3 may not serve as direct target proteins of Mito-ICT-4, while SIRT3 and CypD may serve as direct mitochondrial targets after cellular uptake in the BEL-7402 cell line. Following this, activation of downstream proteins in the p38 MAPK signaling pathway may lead to apoptosis. This hypothesis was further validated by qRT-PCR and Western blot analyses, which demonstrated that Mito-ICT-4 treatment downregulated the expression of SIRT3, p-MKK6, and p-p38 while upregulating the expression of CypD and DDIT3, indicating that these genes may mediate BEL-7402 cells apoptosis induction. Studies have shown [37] that the cascade activation of the P38 MAPK pathway follows an upstream-to-downstream model: stimuli such as redox signals and death domain factors are initially relayed to MAPKKK, then sequentially to MAPKK and MAPK, ultimately influencing gene expression. DDIT3 is among the downstream molecules regulated by MAPK. Consequently, increased intracellular ROS levels may trigger apoptosis by modulating the expression of p-MKK6, p-p38, and DDIT3, thereby activating the p38 MAPK signaling pathway.

## 4. Materials and Methods

### 4.1. Chemistry

Nuclear magnetic resonance (NMR) spectra were obtained using a Varian InfinityPlus-300 spectrometer (Palo Alto, CA, USA) operating at 300 (^1^H) and 75 (^13^C) MHz. The chemical shifts are reported as δ(ppm), and the coupling constants (*J*) are given in Hz for NMR. High-resolution electrospray ionization mass spectrometry (HR-ESI-MS) was conducted using a solanX 70 FT-MS instrument (Bruker, Billerica, MA, USA). Thin layer chromatography (TLC) was performed with precoated silica gel GF-254 glass plates (Qingdao Marine Chemical, Qingdao, China). Icaritin, with a purity above 98%, was purchased from Chengdu Sodium Columbine Lithium Biotechnology Co., Ltd. (Chengdu, China). Synthetic chemicals are purchased from Macklin (Shanghai, China). Solvents of pro-analysis grade were acquired from Xilong Science Co., Ltd. (Shanghai, China).

### 4.2. Synthesis of Intermediates ***1**–**4***

ICT (0.082 mmol, 30 mg), EDCI (0.097 mmol, 18.7 mg), and DMAP (0.073 mmol, 8.9 mg) were added to 8 mL of dichloromethane (DCM), followed by the addition of various bromic acids (0.097 mmol, 17.5 mg/20.2 mg/23.0 mg/25.7 mg). The reaction was conducted in an ice-water bath for 2 h. The reaction completion was monitored by TLC, and a yellow clarified solution was obtained. Water and ethyl acetate were added to the reaction mixture and extracted repeatedly (five times). The organic layer was separated, and the solvent was recovered to yield a powder. The crude product was purified via column chromatography on silica gel (300–400 mesh, petroleum ether: acetone = 15:1–10:1) to yield intermediate products **1**–**4**.

### 4.3. Synthesis of Fatty Acid Analogs ***5**–**9***

ICT (0.082 mmol, 30 mg), EDCI (0.082 mmol, 15.7 mg), and DMAP (0.073 mmol, 8.9 mg) were added to 8 mL of dichloromethane (DCM), followed by the addition of various fatty acids (0.082 mmol, 8.4 mg/10.7 mg/12.9 mg/15.3 mg). The reaction was conducted in an ice-water bath for 1.5 h. The reaction completion was monitored by TLC, resulting in a yellow clarified solution. Water and ethyl acetate were added to the reaction mixture and extracted repeatedly (five times). The organic layer was separated, and the solvent was recovered to yield a powder. The crude product was purified via column chromatography on silica gel (300–400 mesh, petroleum ether: acetone = 10:1) to yield fatty acid analogs **5**–**9**.

### 4.4. Synthesis of Target Compounds Mito-ICT-1~Mito-ICT-4

Intermediates **1**–**4** and TPP^+^ were dissolved in 10 mL of anhydrous acetonitrile (MeCN) at a molar ratio of 1:3.5 and heated to reflux at 85 °C for 60 h. The reaction was basically completed by TLC, and MeCN was removed under reduced pressure. The crude product was purified via column chromatography on silica gel (300–400 mesh, trichloromethane: methanol = 10:1) to yield ICT-TPP^+^-like derivatives Mito-ICT-1–Mito-ICT-4.

### 4.5. Cell Culture

Human cancer cell lines BEL-7402, A549, MCF-7, PC-3M, and normal renal cell line HEK293 were bought from the BeNa Culture Collection (Beijing, China). BEL7402, A549, and PC-3M were cultured in RPMI 1640 medium (Gibco, Carlsbad, CA, USA). MCF-7 and HEK293 were cultured in the DMEM medium (Gibco, CA, USA). The medium was supplemented with 10% fetal bovine serum (FBS) (CLARK, San Diego, CA, USA), 100 U/mL penicillin, and 0.1 mg/mL streptomycin (Beyotime, Nanjing, China). The cells were incubated in 5% CO_2_ at 37 °C.

### 4.6. Detection of Cell Viability by MTT Assay

The MTT assay was employed to assess the inhibitory effect of each compound on cell growth. A549, PC-3M, MCF-7, BEL-7402, and HEK293 cells in the logarithmic growth phase were seeded in 96-well culture plates at an inoculum density of 5 × 10^4^ cells/mL. After overnight incubation at 37 °C in a 5% CO_2_ atmosphere, the cells fully adhered to the surface. Different concentrations of sample solutions (0, 3.125, 6.25, 12.5, 25, 50, 100 μmol/L) were added (100 μL) to treat the cells. After 48 h of drug treatment, 10 μL of a 5 mg/mL MTT solution was added to each well and incubated for 4 h at 37 °C. The supernatant was then discarded, and 150 μL of DMSO was added to dissolve the formazan. The mixture was shaken at room temperature for 15 min, and the absorbance (D) value was measured at 490 nm using a Microplate Reader (HBS-ScanY, Nanjing, China). The drug IC50 values were calculated using GraphPad Prism (v10.0). Each cell viability experiment was performed in triplicate.

### 4.7. HPLC Analysis of Cellular Uptake of Compounds

BEL-7402 cells were cultured in 100 mm Petri dishes. When the cell density reached 90%, the cells were treated with different drugs, all at the same concentration (ICT and Mito-ICT-4 were both set at 10 μM). After 9 h of drug treatment, intracellular mitochondria were isolated from the different drug-treated groups using a mitochondrial isolation kit (Beyotime, Nanjing, China). The mitochondria were resuspended in 200 μL of phosphate-buffered saline (PBS), and an equal volume of anhydrous dichloromethane was added. Ultrasonic extraction was performed three times, and the lower organic phase was collected. Dichloromethane was removed, and the sample was resuspended in 200 μL of 0.1% formic acid in methanol. A 20 μL sample was analyzed and quantified by HPLC. HPLC analysis was conducted using an ALLTECH 26-201 (Jerome, CA, USA) with a flow rate of 1 mL/min. The stationary phase was Agilent ZORBAX SB-C18 (250 mm × 4.6 mm, 5 μm, Santa Clara, CA, USA), and the mobile phase consisted of methanol-0.1% formic acid in water (80:20).

### 4.8. Apoptosis Was Observed Using DAPI Staining

BEL-7402 cells in the logarithmic growth phase were seeded in 12-well plates at a density of 1 × 10^5^ cells/well. After overnight incubation, the cells were treated with different concentrations of Mito-ICT-4 (0.625, 1.25, and 2.5 μmol/L), and a control group was included. After 48 h of incubation, the cells were fixed with 4% histocyte fixative (Solarbio, Beijing, China) at room temperature for 15 min. After fixation, 500 μL of DAPI staining solution (Solarbio, Beijing, China) was added to each well and incubated at 37 °C for 20 min. The cells were then observed under an inverted fluorescence microscope (EVOS M5000, Waltham, MA, USA).

### 4.9. Apoptosis Was Detected Using Annexin V-FITC/PI Double Staining

BEL-7402 cells in the logarithmic growth phase were prepared into a cell suspension at a concentration of 2.5 × 10^5^ cells/mL and seeded into 6-well culture plates with 2 mL per well. After overnight incubation, different concentrations of Mito-ICT-4 (0.625, 1.25, 2.5 μmol/L) were added to the cells, and a control group was included. After 48 h of culture, the supernatant was transferred to a centrifuge tube. The cells were then digested with pancreatin (without EDTA) (Beyotime, Nanjing, China) at room temperature and centrifuged at 4 °C for 5 min (10,000 rpm). The supernatant was discarded. Then, 300 μL of 1× buffer was added to each group, and the mixture was well mixed. The mixture was then incubated with 5 μL of Annexin V-FITC (Solarbio, Beijing, China) solution at room temperature for 5 min, protected from light, followed by the addition of 5 μL of PI solution. Apoptosis was detected using flow cytometry (BD Biosciences, Franklin Lakes, NJ, USA).

### 4.10. Mitochondrial Membrane Potential Was Detected Using JC-1 Staining

Drug grouping and administration were performed as described in Section 2.5. After 48 h of incubation, 500 μL of JC-1 fluorescent dye (Beyotime, Nanjing, China) was added to each well. The cells were stained at 37 °C for 30 min, after which the dye was discarded. The cells were then washed three times with PBS and observed under an inverted fluorescence microscope.

### 4.11. Calcein AM Probe Detects MPTP Opening

Drug grouping and administration were performed as described in Section 2.5. After 24 h of incubation, 500 μL of staining solution (containing 2 μM Calcein AM and 5 mM CoCl_2_) (Aladdin, Shanghai, China) was added to each well. The cells were stained for 30 min at 37 °C and protected from light. After staining, the solution was discarded, and the cells were washed twice with PBS. The cells were then observed under an inverted fluorescence microscope.

### 4.12. Molecular Docking

The crystal structures of SIRT3, CypD, MKK6, P-38, and DDIT3 were first obtained from the Protein Data Bank (PDB) (http://www.rcsb.org, accessed on 13 September 2023). Water molecules and redundant complexes were removed using PyMOL (v3.0.3) software. Subsequently, the small molecule ligands were visualized using ChemDraw (v22.0.0) and Chem3D (v14.0.0.17), and the molecular ligands of the compounds were then docked with the target protein receptors. The molecular ligands of the compounds were docked with the target protein receptors via molecular simulation, and the results were visualized and analyzed using PyMOL and LigPlot (v2.2.9) software.

### 4.13. DCFH-DA Fluorescent Probe to Detect ROS Levels

Drug grouping and administration were performed as described in Section 2.6. After 48 h of incubation, 500 μL of DCFH-DA probe (Beyotime, Nanjing, China) was added to each well. The cells were stained for 30 min at 37 °C and protected from light. The dye was then discarded, and the cells were washed three times with a serum-free medium. Samples were analyzed using flow cytometry with an excitation wavelength of 480 nm and an emission wavelength of 525 nm.

### 4.14. Transcriptome Sequencing

After 48 h of treatment with Mito-ICT-4, the cells were collected, and a blank group was established. A volume of 1 mL of Trizol (CWBIO, Beijing, China) was added to each sample, and total RNA was extracted to prepare a cell suspension for transcriptome sequencing, which was performed with the assistance of Sangon Biotech (Shanghai, China). Gene differential expression analysis was conducted using DESeq2 (v1.26.0) software, applying the criteria of |log2FC| ≥ 1 and *p*-value < 0.05 to screen for differentially expressed genes. These genes were further analyzed for enrichment using Gene Ontology (GO) (http://www.geneontology.org, accessed on 20 October 2023) and KEGG (http://www.kegg.jp, accessed on 20 October 2023).

### 4.15. qRT-PCR Validation of Differential Genes

Total RNA was extracted from BEL-7402 cells before and after Mito-ICT-4 treatment using the Trizol method. The RNA was then reverse transcribed and used for quantitative real-time PCR (qRT-PCR) analysis. All gene sequences were obtained from the official website of NCBI (http://ncbi.nlm.nih.gov, accessed on 16 December 2023), and primers were designed and synthesized by Sangon Biotech. β-actin was used as an internal reference. To each well, 10 µL of 2 × SGExcel FastSYBR (Sangon Biotech, Shanghai, China) mixture was added, along with 0.4 µL each of forward and reverse primers (F/R), followed by 2 µL of cDNA. The volume was then adjusted to 20 µL with water. The qRT-PCR amplification procedure included the following steps: pre-denaturation at 95 °C for 3 min, denaturation at 95 °C for 15 s, and annealing at 60 °C for 45 s. A total of 45 cycles were performed. Fluorescence signals were collected from 60 °C to 95 °C using an ABI 7500 fluorescence quantitative PCR instrument (ABI, Waltham, MA, USA). Gene expression was calculated using the 2^−ΔΔCt^ method.

### 4.16. Western Blot

Drug grouping and administration were performed as described in Section 2.6. After 48 h of incubation, the supernatant was discarded, and the cells were washed twice with pre-cooled PBS. A volume of 150 µL of RIPA lysis buffer (Solarbio, Beijing, China) was added and incubated on ice. After sufficient lysis, the supernatant was centrifuged, and the protein concentration was determined using a BCA kit (Thermo, Waltham, MA, USA). The SDS-PAGE gel system was chosen based on the molecular weight of the target proteins to perform electrophoresis. After electrophoresis, the processed samples were transferred to a membrane and incubated at room temperature for 2 h to block non-specific binding. After membrane transfer, the membranes were incubated with primary antibodies against CypD (Proteintech Group, Wuhan, China), SIRT3 (Abcam, Cambridge, UK), P-MKK6 (Bioss, Beijing, China), P-P38 (Bioss, Beijing, China), DDIT3 (Bioss, Beijing, China), Cleaved-caspase-3 (Abcam, Cambridge, UK), and β-actin (ZSGB-BIO, Beijing, China) (1:1000) at 4 °C overnight. After primary antibody binding, the membranes were incubated with the corresponding secondary antibody (ZSGB-BIO, Beijing, China) (1:3000) at room temperature for 1 h. Immunoblot bands were detected using an enhanced chemiluminescence system (Analytik Jena AG, Jena, Germany). The bands were analyzed using ImageJ (v1.8.0) software.

## 5. Conclusions

The effective treatment of tumors remains a global medical challenge. Currently, the primary obstacles associated with antitumor drugs include significant toxic side effects and inadequate targeting. While targeting tumor tissues, these drugs not only eradicate tumor cells but also damage normal cells, resulting in severe toxic effects. Consequently, the development of highly targeted therapies for tumor treatment remains a central focus in oncology. Conjugates based on TPP^+^ have been widely utilized to target the mitochondria of tumor cells, thereby enhancing the antitumor activity and cellular selectivity of these compounds. In this study, we developed an optimized synthesis strategy for ICT derivatives and synthesized a series of mitochondria-targeted ICT derivatives with high yields. Through activity screening assays, we demonstrated that TPP+ serves as a reliable mitochondria-targeted cationic carrier, significantly enhancing antitumor activity and selectivity for normal cells upon binding with ICT. Among these derivatives, Mito-ICT-4 exhibited the strongest antiproliferative effect on BEL-7402 cells, with an IC_50_ value as low as 0.73 ± 0.06 μM. HPLC results demonstrated that Mito-ICT-4 significantly accumulated in the mitochondria of BEL-7402 cells, with the level of accumulation approximately 2.5 times higher than that of ICT. Further studies revealed that, upon targeting the mitochondria of tumor cells, Mito-ICT-4 downregulated SIRT3 protein expression, disrupted intracellular redox homeostasis, and markedly increased mitochondrial ROS levels. Additionally, it facilitated the opening of CypD-dependent MPTP, induced mitochondrial membrane potential depolarization, and triggered ROS release into the cytoplasm, which activated the ROS-mediated p38 MAPK signaling pathway, ultimately leading to mitochondria-dependent apoptosis in BEL-7402 cells.

## Figures and Tables

**Table 1:** Antiproliferative activity of ICT derivatives against various cell lines.

Compound	Compounds IC_50_ Values (μM) ^a^				
	BEL-7402	MCF-7	A549	PC-3M	HEK293
**1**	47.83 ± 2.65	25.78 ± 1.07	35.84 ± 3.57	44.52 ± 2.15	46.82 ± 1.82
**2**	40.59 ± 5.50	42.05 ± 5.47	>100	76.13 ± 4.44	>100
**3**	>100	>100	>100	>100	>100
**4**	>100	>100	>100	76.04 ± 4.69	>100
**5**	39.88 ± 2.06	48.92 ± 1.42	97.81 ± 6.40	60.25 ± 3.88	77.17 ± 5.13
**6**	40.56 ± 5.17	45.58 ± 3.02	>100	>100	>100
**7**	74.57 ± 4.83	>100	>100	>100	>100
**8**	>100	>100	>100	>100	>100
Mito-ICT-1	2.85 ± 0.70	10.70 ± 0.35	21.11 ± 2.67	33.57 ± 2.53	47.01 ± 2.91
Mito-ICT-2	3.43 ± 0.91	23.68 ± 2.24	10.72 ± 1.09	27.83 ± 1.74	53.72 ± 5.15
Mito-ICT-3	3.85 ± 0.69	22.46 ± 1.78	13.68 ± 0.92	20.61 ± 2.06	54.99 ± 3.92
Mito-ICT-4	0.73 ± 0.06	10.79 ± 1.20	5.15 ± 0.59	11.26 ± 1.55	67.11 ± 2.09
ICT	21.78 ± 2.42	25.72 ± 1.89	43.09 ± 1.93	63.61 ± 2.67	43.19 ± 3.05
DOX	3.23 ± 0.64	2.44 ± 0.81	6.88 ± 0.62	0.24 ± 0.11	>100
5-FU	38.96 ± 1.32	13.45 ± 2.78	86.21 ± 7.94	58.67 ± 2.64	>100

^a^ Cell viability was assessed following 48 h of drug exposure to the cells. All experimental results were replicated at least three times. The data are expressed as the mean ± standard deviation.

**Table 2:** Molecular docking results.

Compound	Affinity/kcal·mol^−1^				
	SIRT3	CypD	MKK6	P-38	DDIT3
ICT	−9.5	−7.9	−10.3	−9.0	−7.0
Mito-ICT-4	−13.7	−11.8	−5.7	−7.9	−5.8

## Data Availability

The data that support the findings of this study are available from the corresponding author upon reasonable request.

## Supplementary Materials

The following supporting information can be downloaded at https://www.mdpi.com/article/10.3390/molecules30081667/s1. BEL-7402 cell STR identification report; Figures S1–S12: NMR spectra of compounds; Figures S13–S19: Full image of the original uncropped Western blot in this study; Table S1: Primers used in this study.
