# Supplementary material for: The Mechanism of a Novel Mitochondrial-Targeted Icaritin Derivative in Regulating Apoptosis of BEL-7402 Cells Based on the SIRT3 and CypD-Mediated ROS/p38 MAPK Signaling Pathway
Source: Molecules. 2025 Apr 8;30(8):1667. doi: 10.3390/molecules30081667 (PMC12029982; doi:10.3390/molecules30081667)

Applied Biosystems
GeneMapper 4.0

Cell Line Authentication-12

Sample File	Sample Name	Panel	OS	SQ
83_C11_CellLineAuthentication-0419.fsa	Bel-7402	STR Profile 1-human		

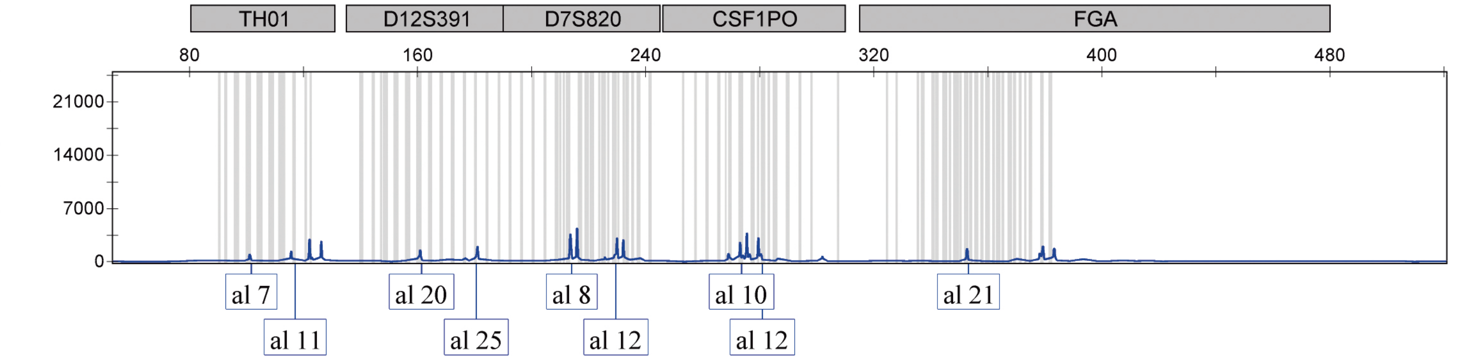

Sample File	Sample Name	Panel	OS	SQ
96_H12_CellLineAuthentication-0414.fsa	Bel-7402	STR Profile 2-human		

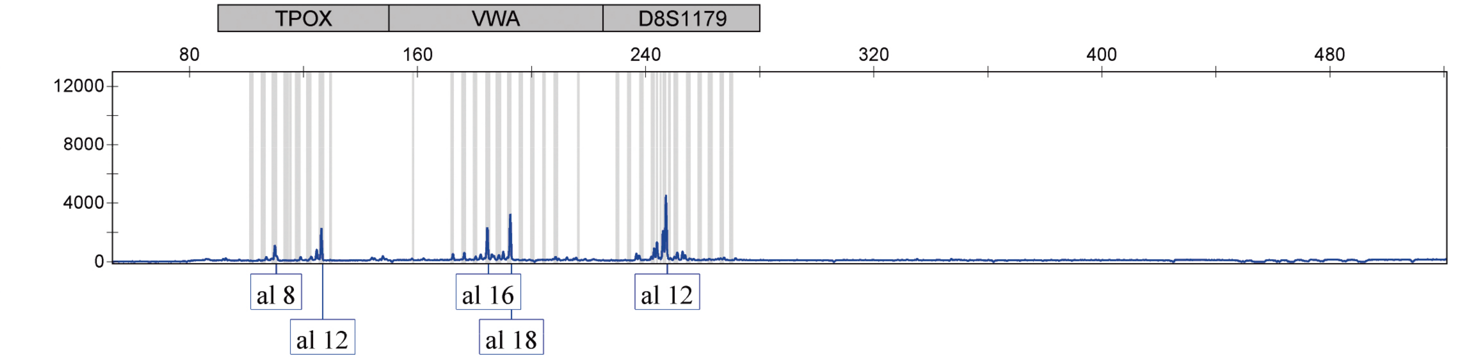

Sample File	Sample Name	Panel	OS	SQ
96_H12_CellLineAuthentication-0419.fsa	Bel-7402	STR Profile 3-human		

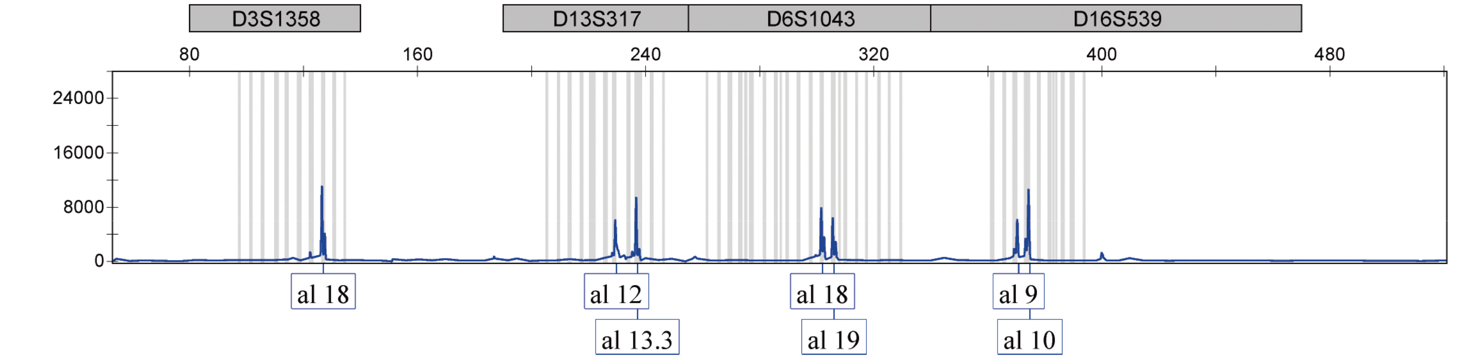

Sample File	Sample Name	Panel	OS	SQ
32_H04_CellLineAuthentication-0414.fsa	Bel-7402	STR Profile 4-human		

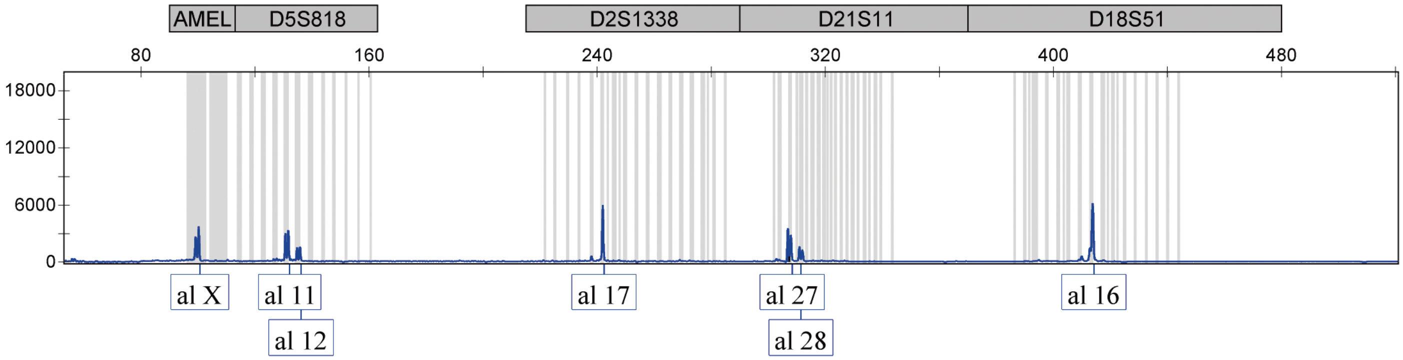

Cell Line Authentication-12

Sample File	Sample Name	Panel	OS	SQ
72_H09_CellLineAuthentication-0414.fsa	Bel-7402	STR Profile 1-human		

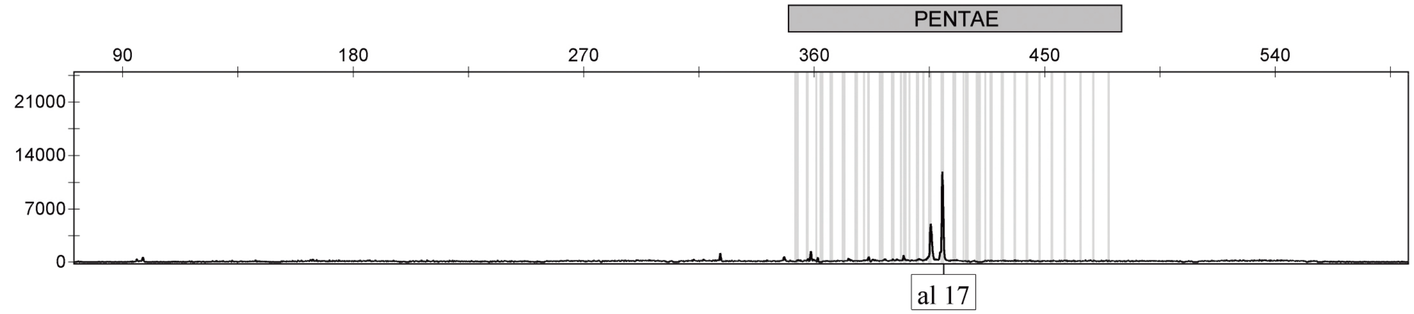

Sample File	Sample Name	Panel	OS	SQ
96_H12_CellLineAuthentication-0414.fsa	Bel-7402	STR Profile 2-human		

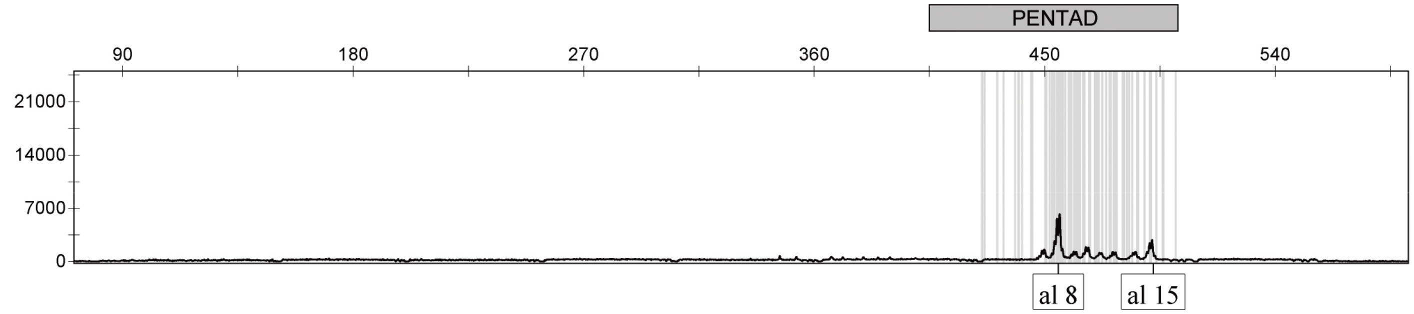

Sample File	Sample Name	Panel	OS	SQ
96_H12_MingQingSenMAOA-2(2-9)1-8-0414.fsa	Bel-7402	STR Profile 3-human		

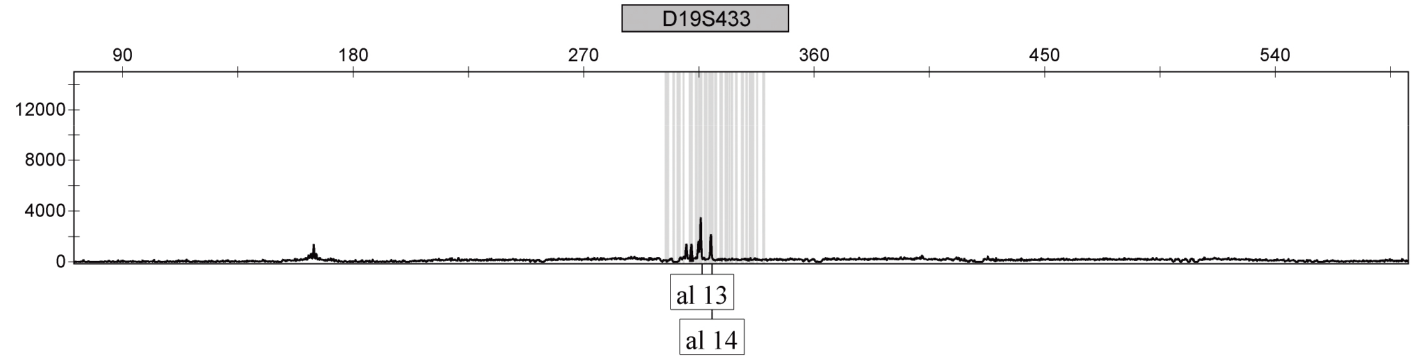

Supplement: Supplementary file 1 [file molecules-30-01667-s001.zip › Bel-7402(STR identification data results).pdf]
