# Supplementary material for: The Mechanism of a Novel Mitochondrial-Targeted Icaritin Derivative in Regulating Apoptosis of BEL-7402 Cells Based on the SIRT3 and CypD-Mediated ROS/p38 MAPK Signaling Pathway
Source: Molecules. 2025 Apr 8;30(8):1667. doi: 10.3390/molecules30081667 (PMC12029982; doi:10.3390/molecules30081667)
Supplement: Supplementary file 1 [file molecules-30-01667-s001.zip › Supplemental.pdf]

Figure. S1 Compound 1 ^{1}H NMR (300MHz, $CDCl_3$).

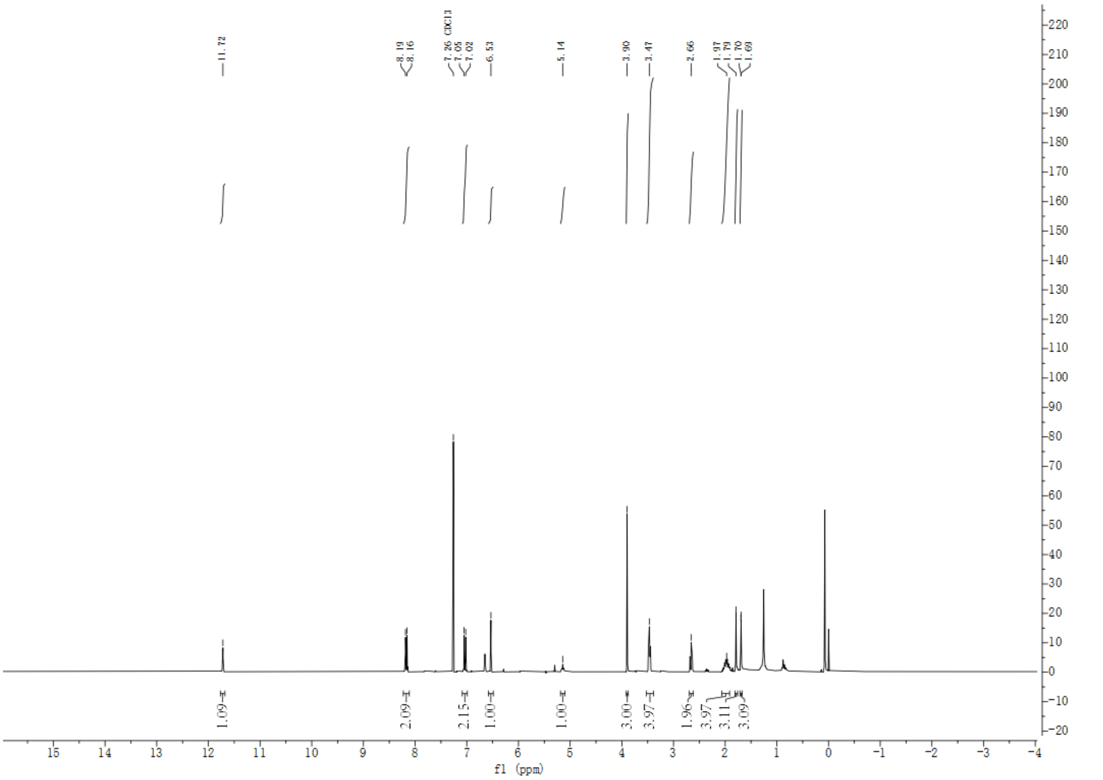

Figure. S1 Compound 1 ^{13}C NMR (75MHz, $CDCl_3$).

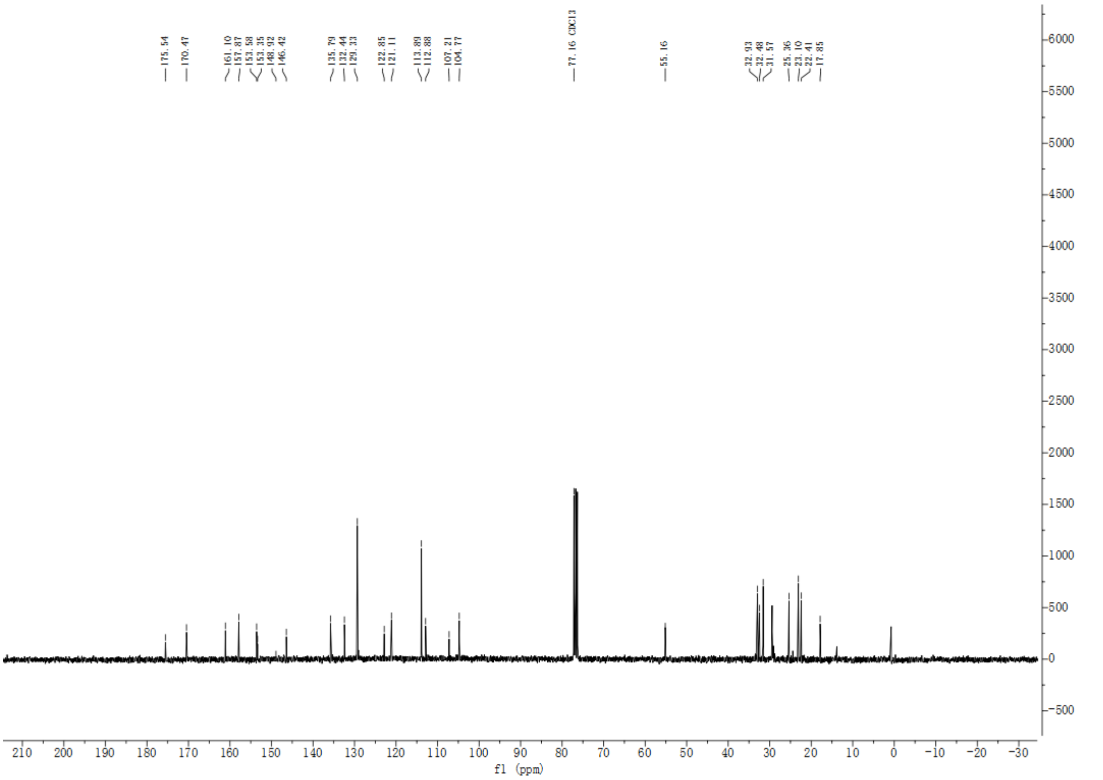

Figure. S2 Compound 2 ^{1}H NMR (300MHz, $CDCl_3$).

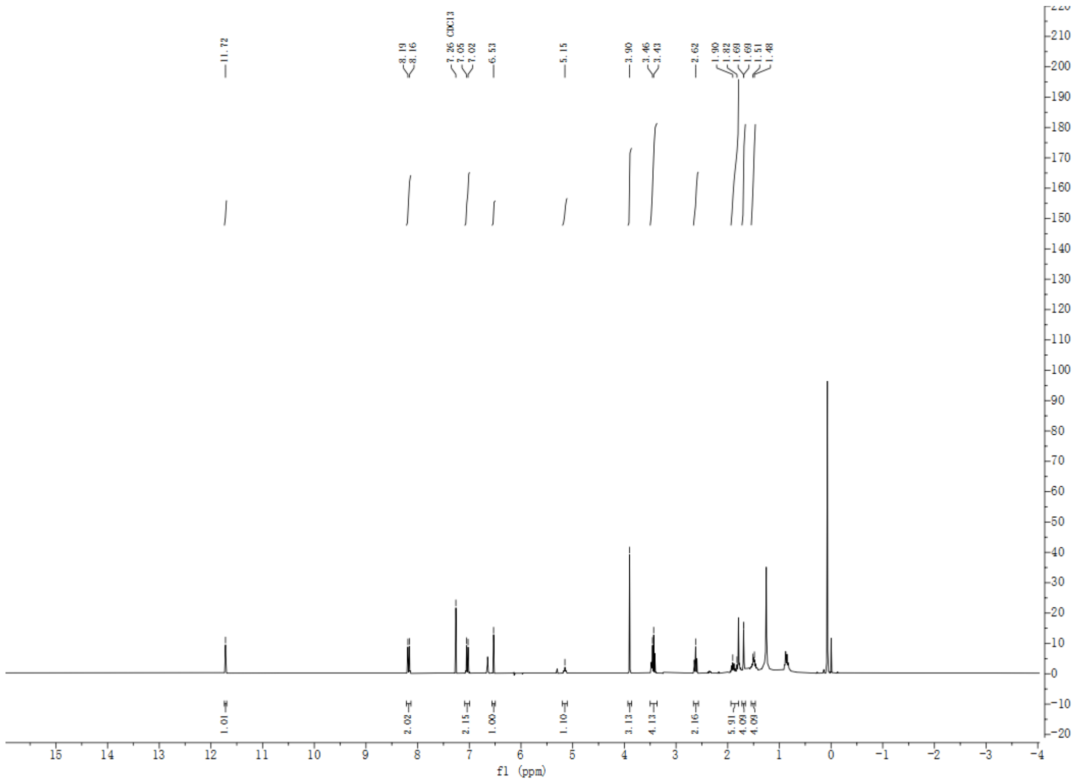

Figure. S2 Compound 2 ^{13}C NMR (75MHz, $CDCl_3$).

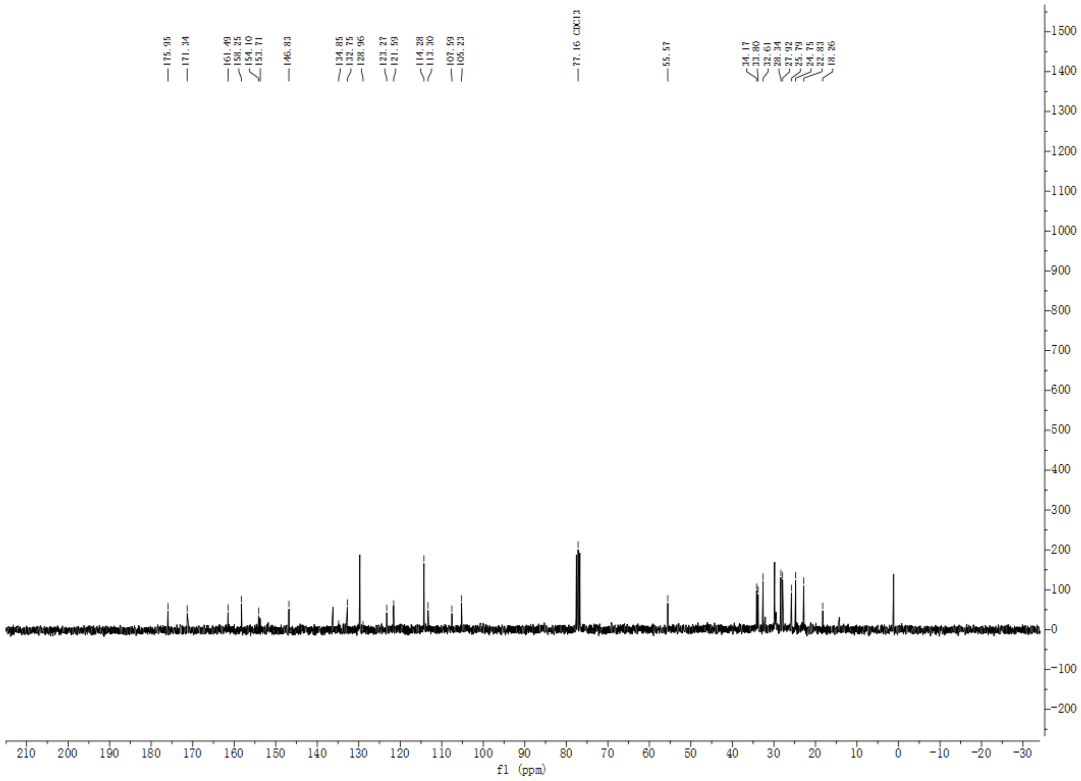

Figure. S3 Compound 3 ^{1}H NMR (300MHz, $CDCl_3$).

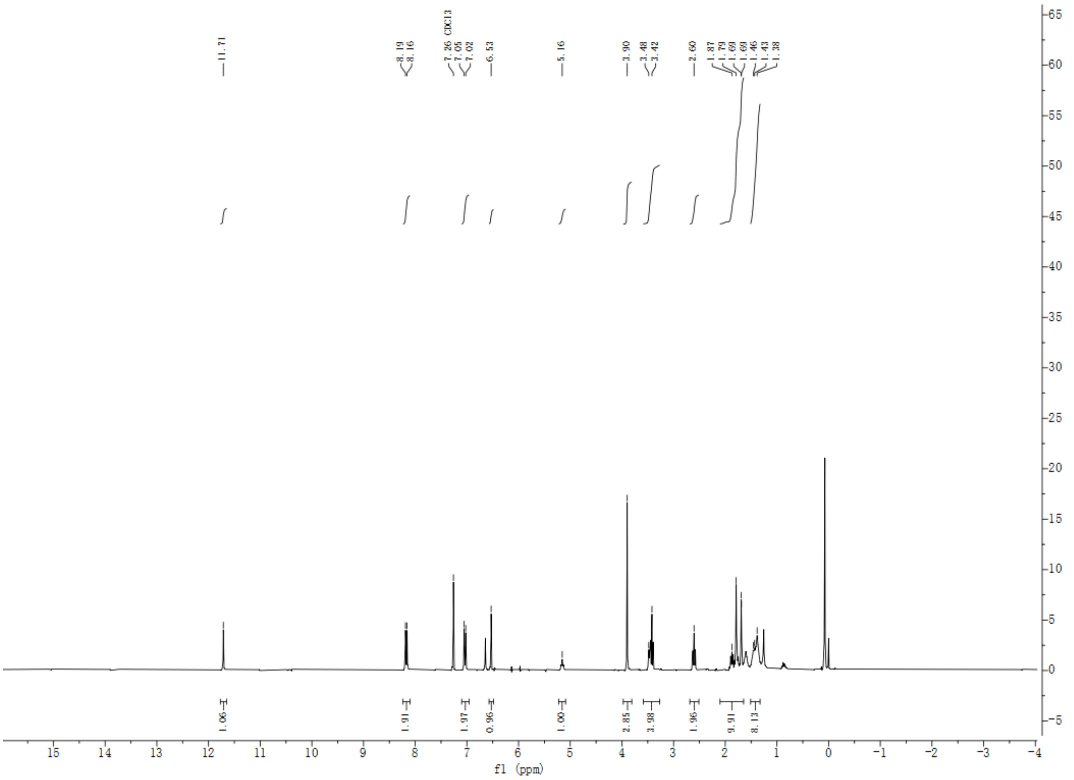

Figure. S3 Compound 3 ^{13}C NMR (75MHz, $CDCl_3$).

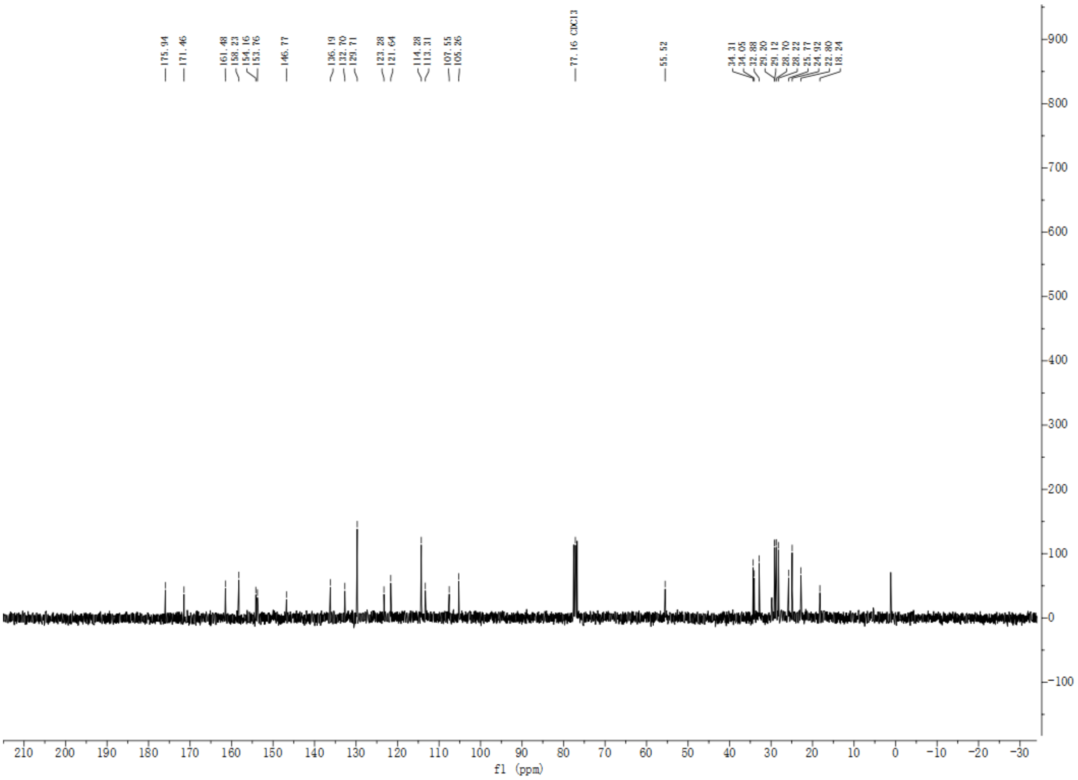

Figure. S4 Compound 4 ^{1}H NMR (300MHz, $CDCl_3$).

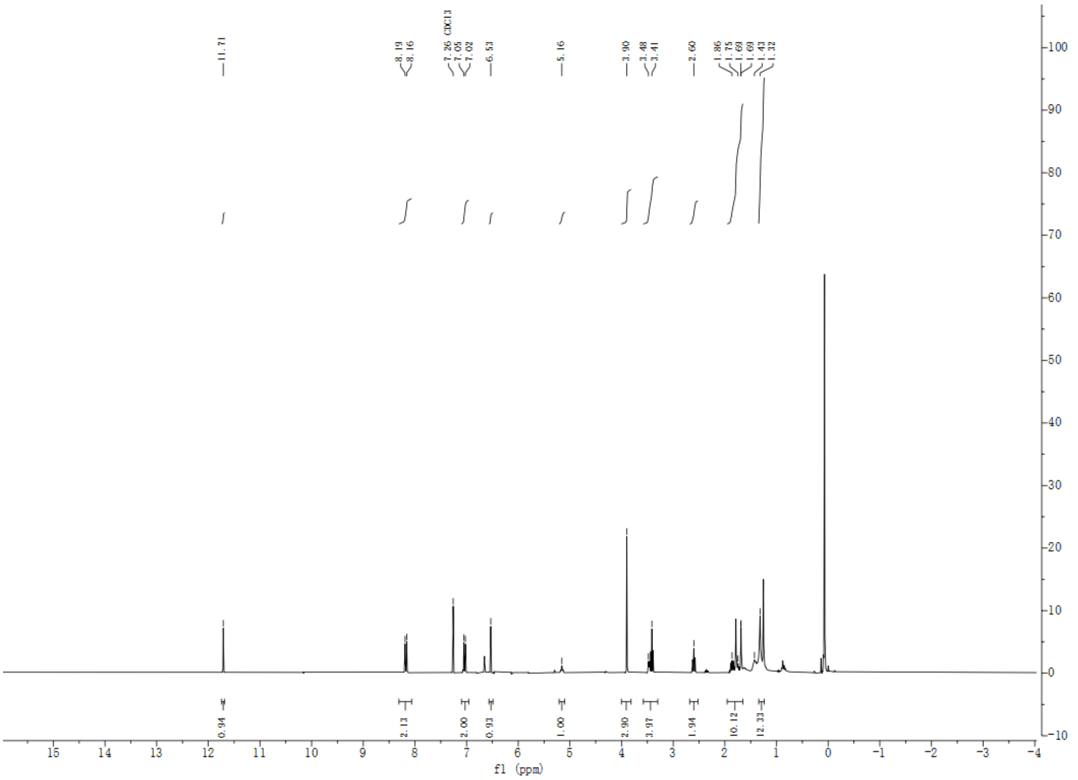

Figure. S4 Compound 4 ^{13}C NMR (75MHz, $CDCl_3$).

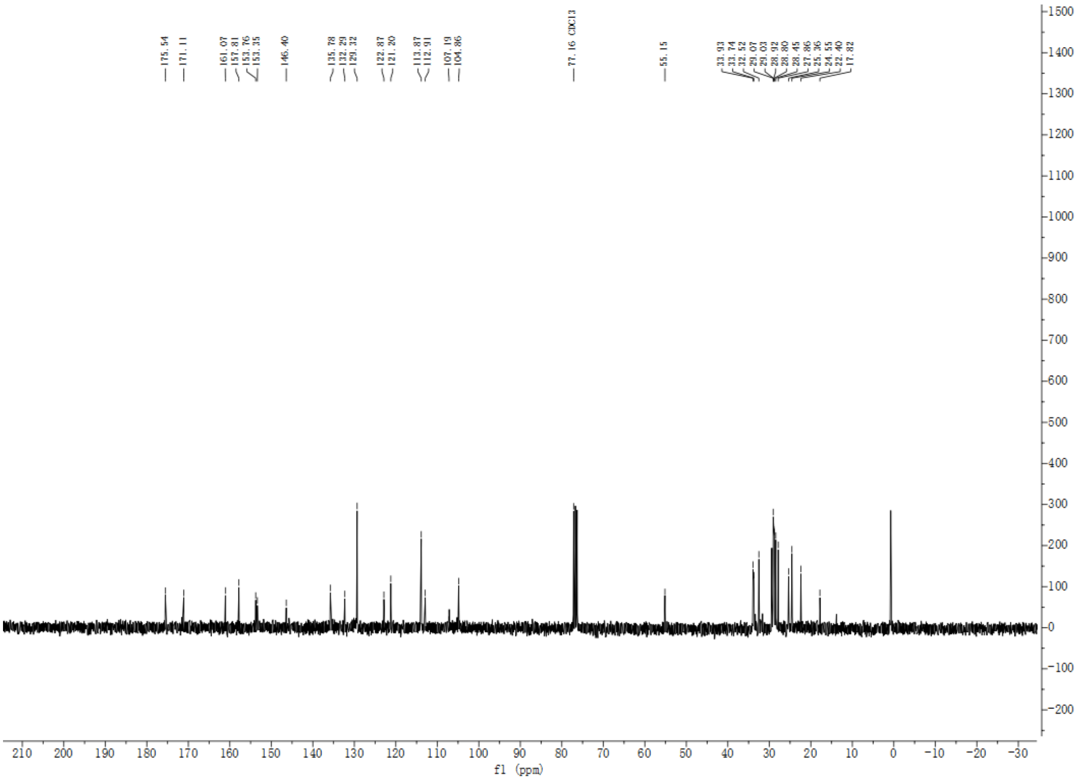

Figure. S5 Compound 5 ^{1}H NMR (300MHz, $CDCl_3$).

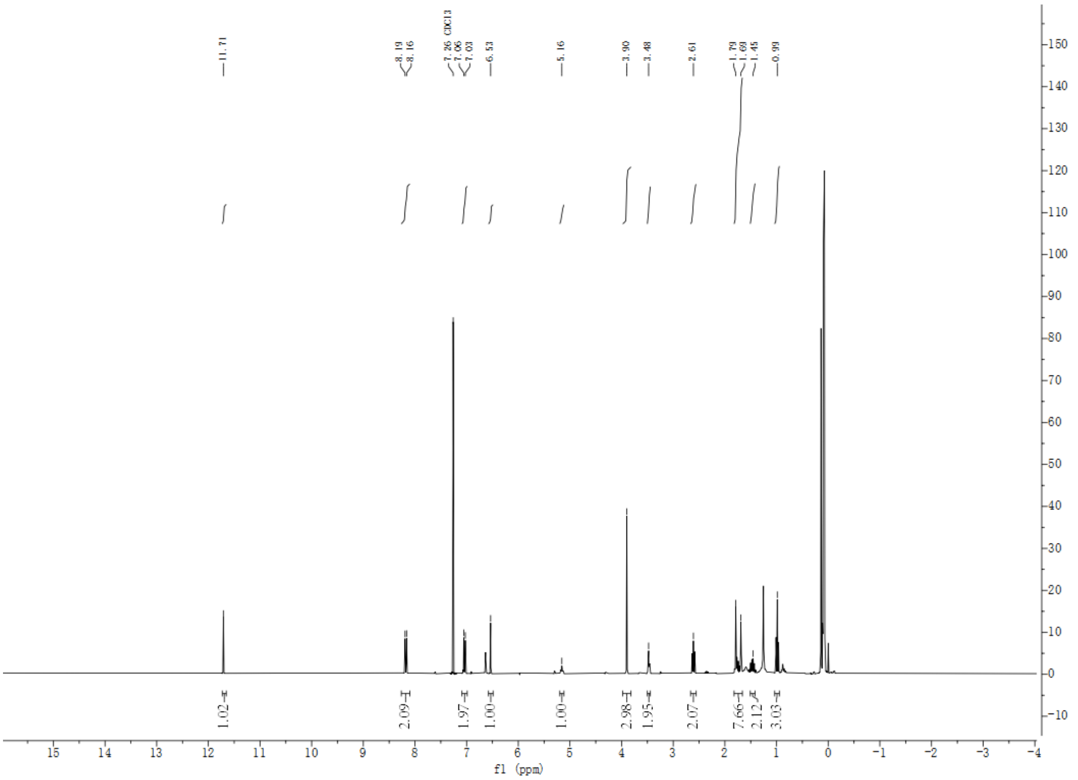

Figure. S5 Compound 5 ^{13}C NMR (75MHz, $CDCl_3$).

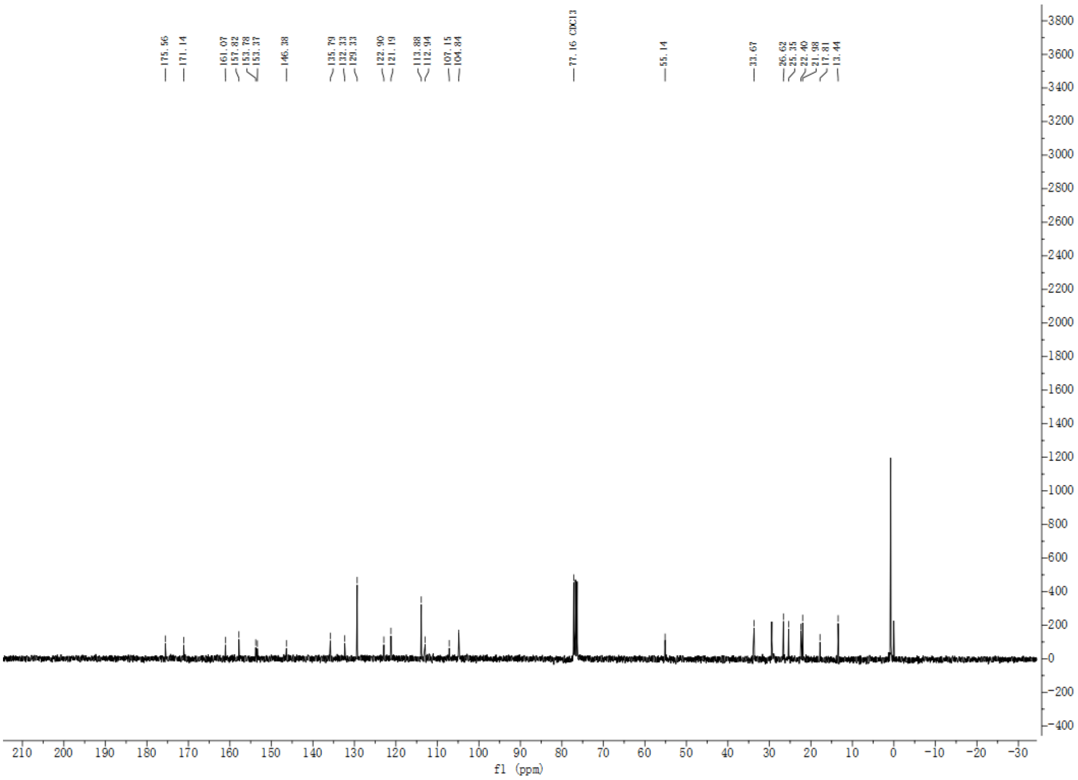

Figure. S6 Compound 6 ^{1}H NMR (300MHz, $CDCl_3$).

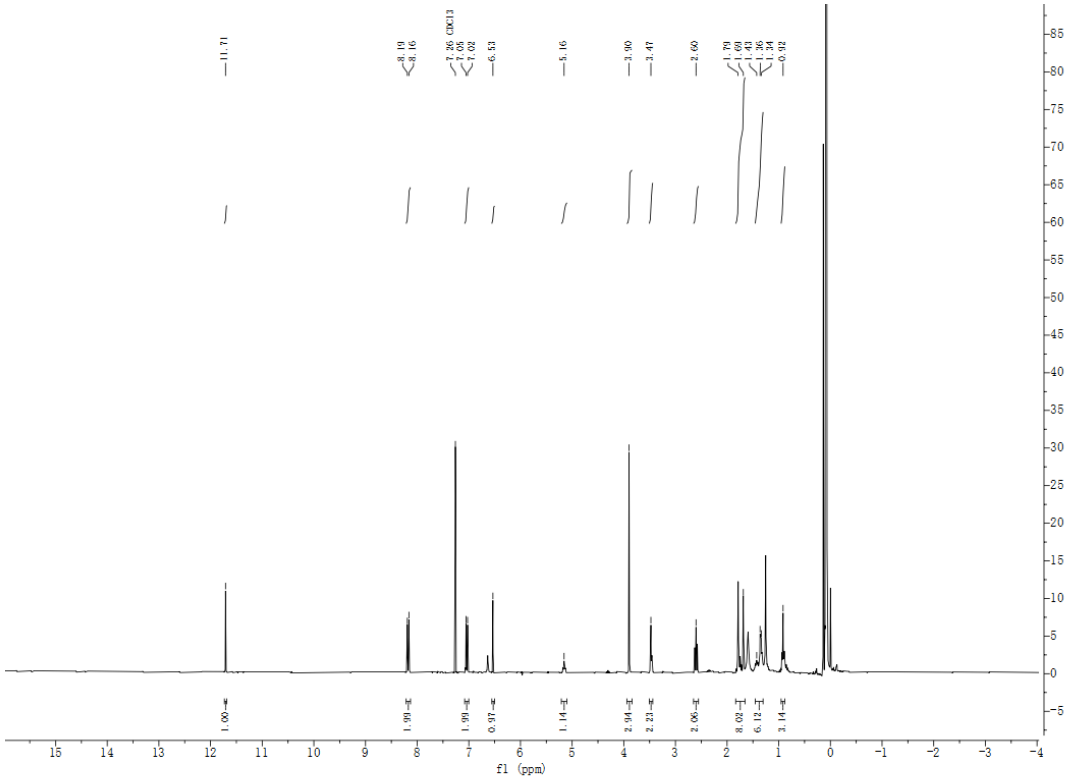

Figure. S6 Compound 6 ^{13}C NMR (75MHz, $CDCl_3$).

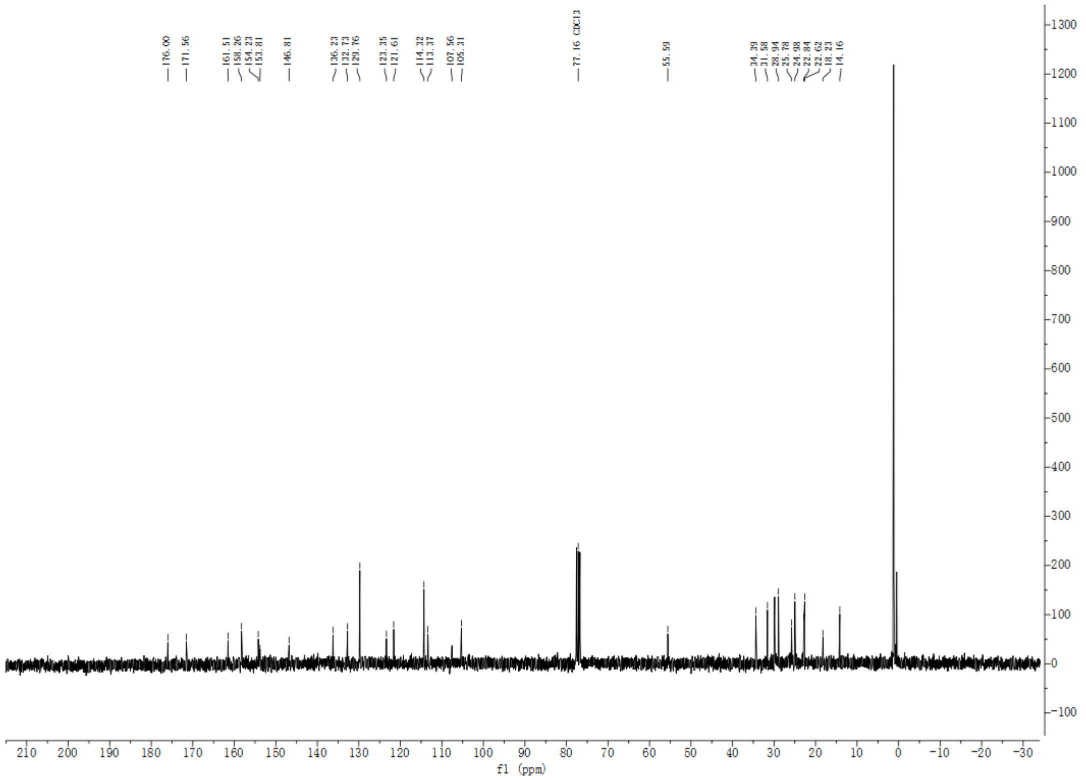

Figure. S7 Compound 7 ^{1}H NMR (300MHz, CDCl3).

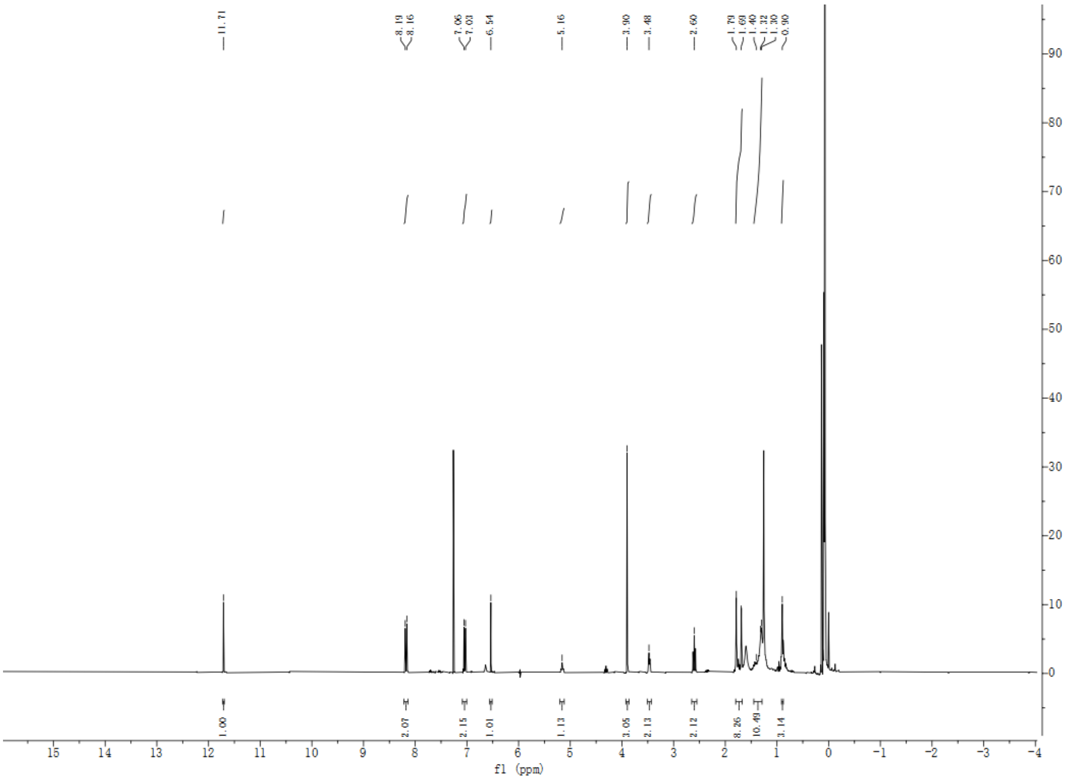

Figure. S7 Compound 7 ^{13}C NMR (75MHz, CDCl3).

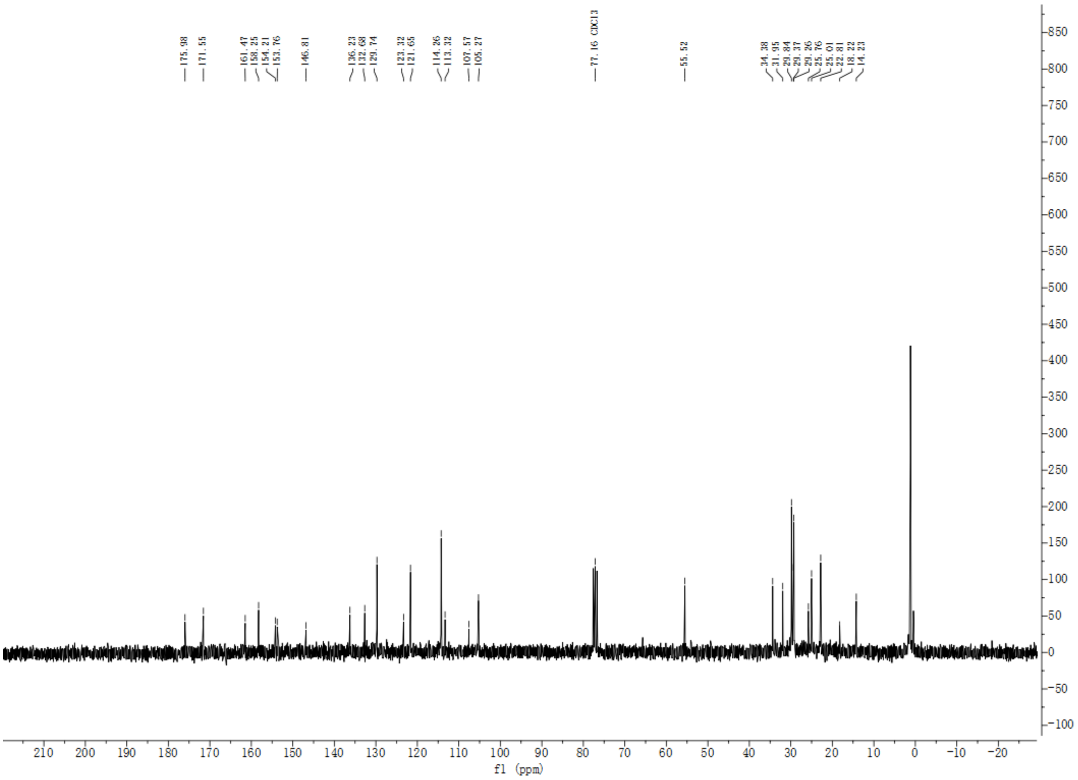

Figure. S8 Compound 8 ^{1}H NMR (300MHz, $CDCl_3$).

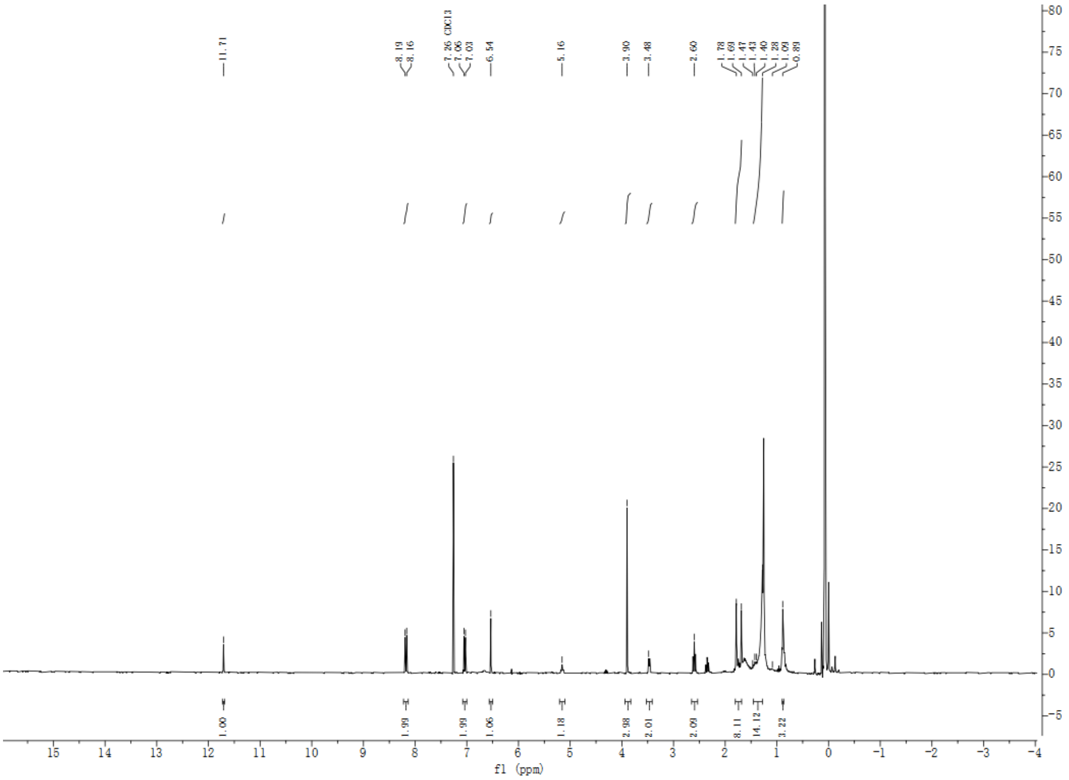

Figure. S8 Compound 8 ^{13}C NMR (75MHz, $CDCl_3$).

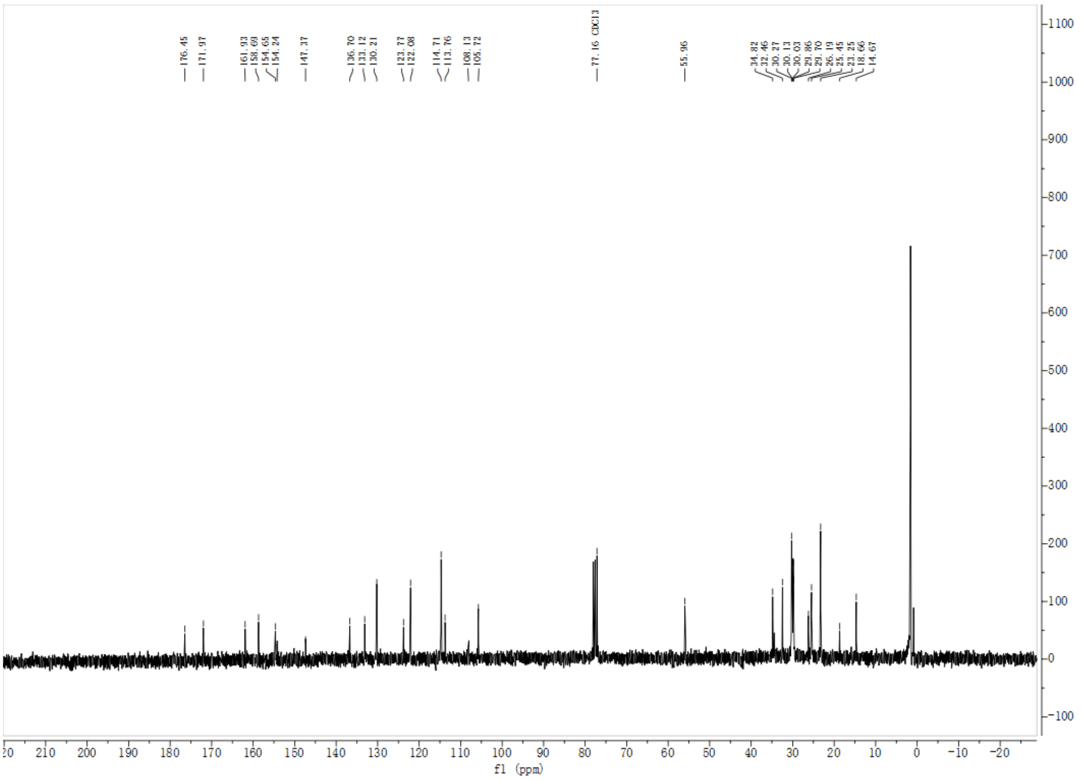

Figure. S9 Compound Mito-ICT-1 ^{1}H NMR (300MHz, $CDCl_3$).

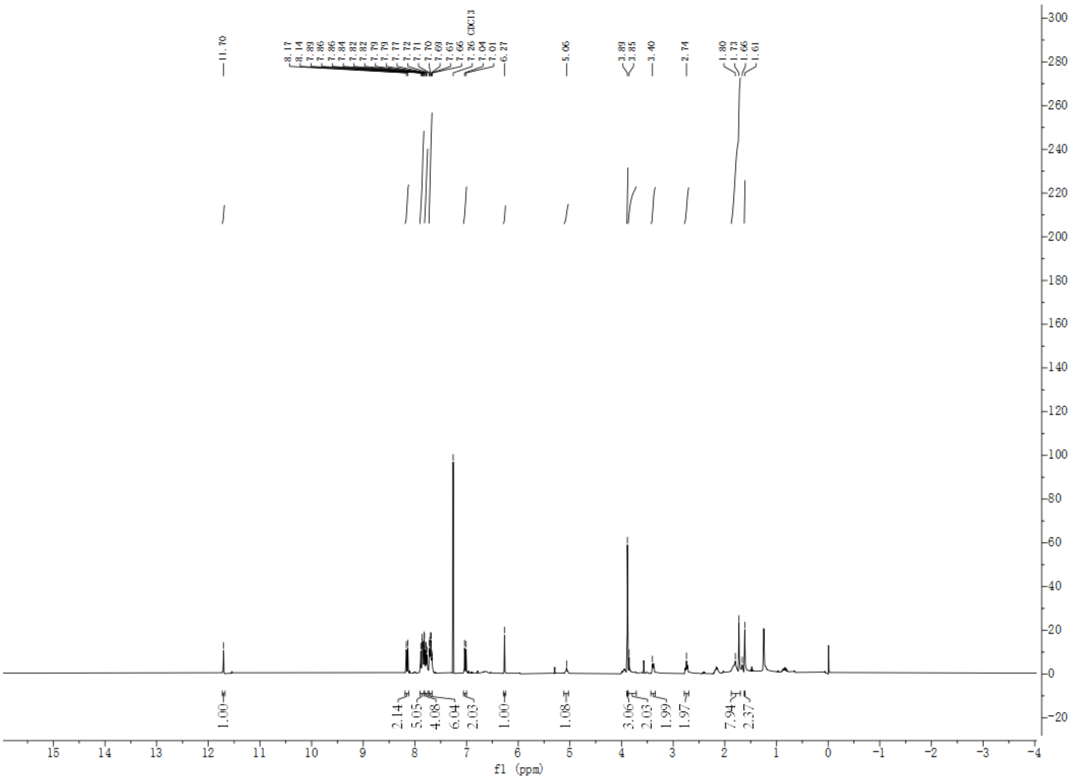

Figure. S9 Compound Mito-ICT-1 ^{13}C NMR (75MHz, $CDCl_3$).

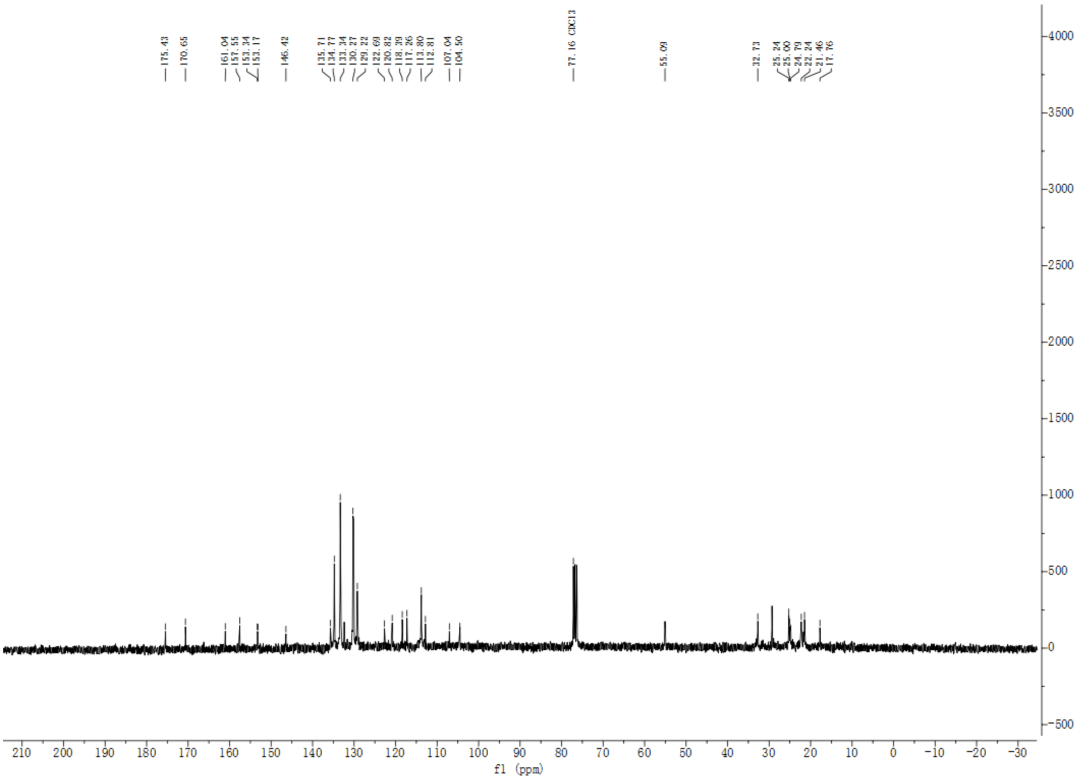

Figure. S10 Compound Mito-ICT-2 ^{1}H NMR (300MHz, $CDCl_3$).

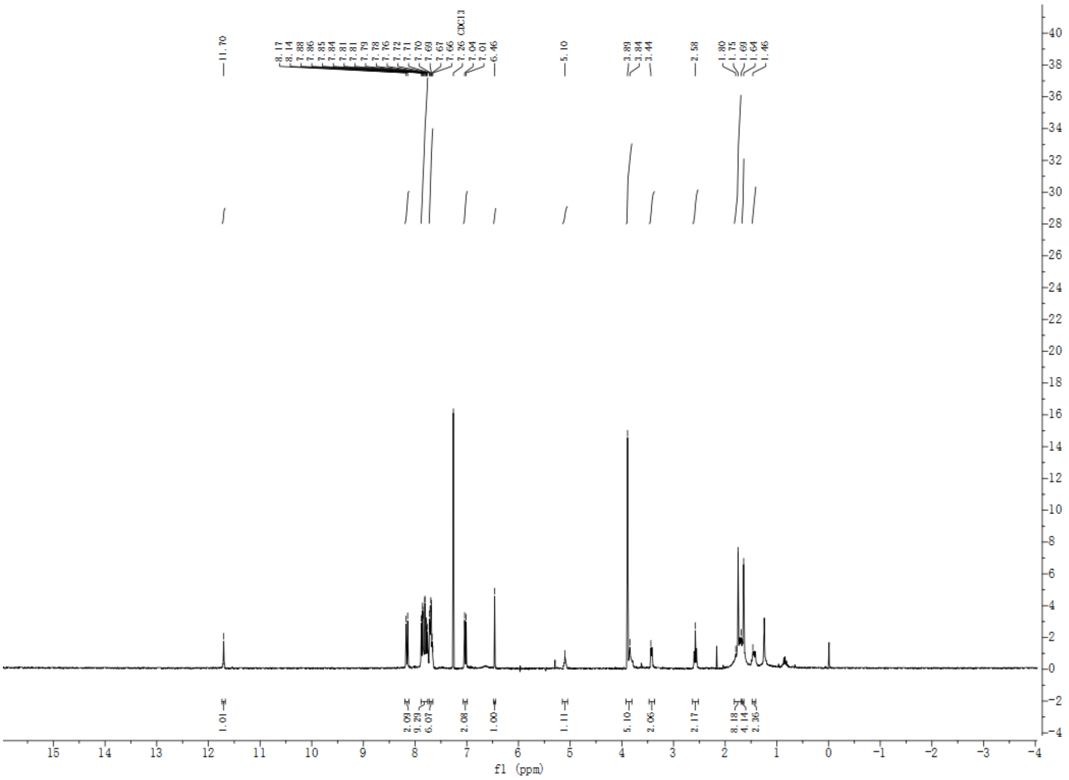

Figure. S10 Compound Mito-ICT-2 ^{13}C NMR (75MHz, $CDCl_3$).

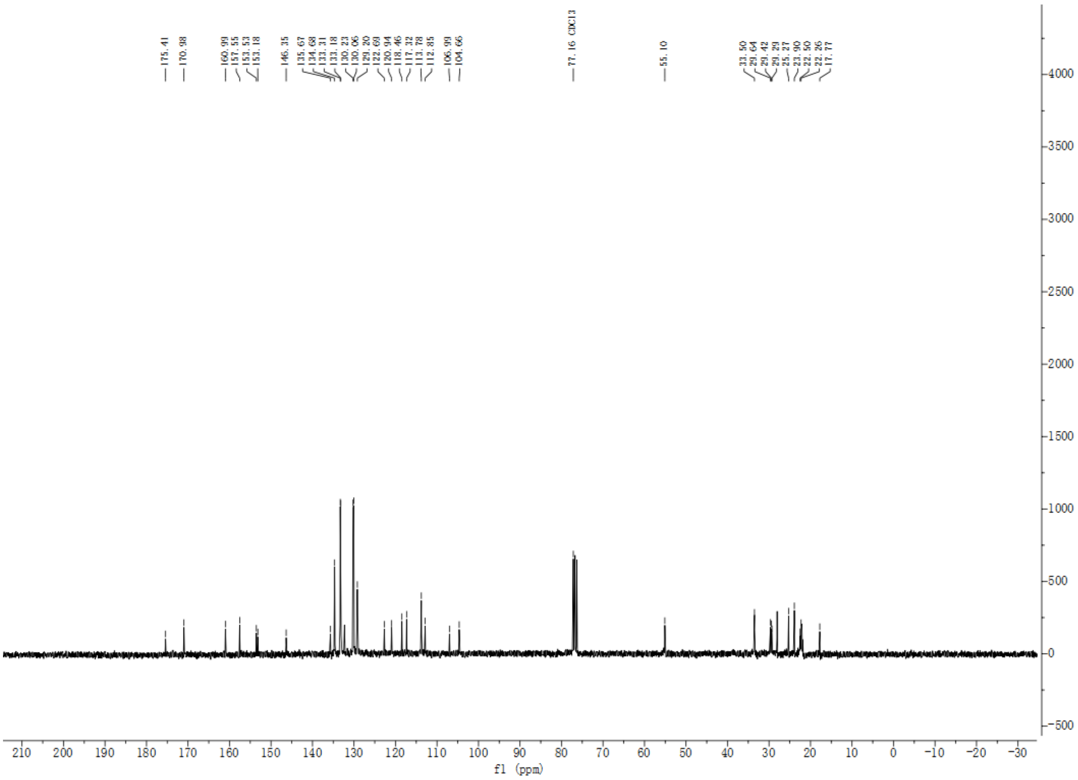

Figure. S11 Compound Mito-ICT-3 ^1H NMR (300MHz, $CDCl_3$).

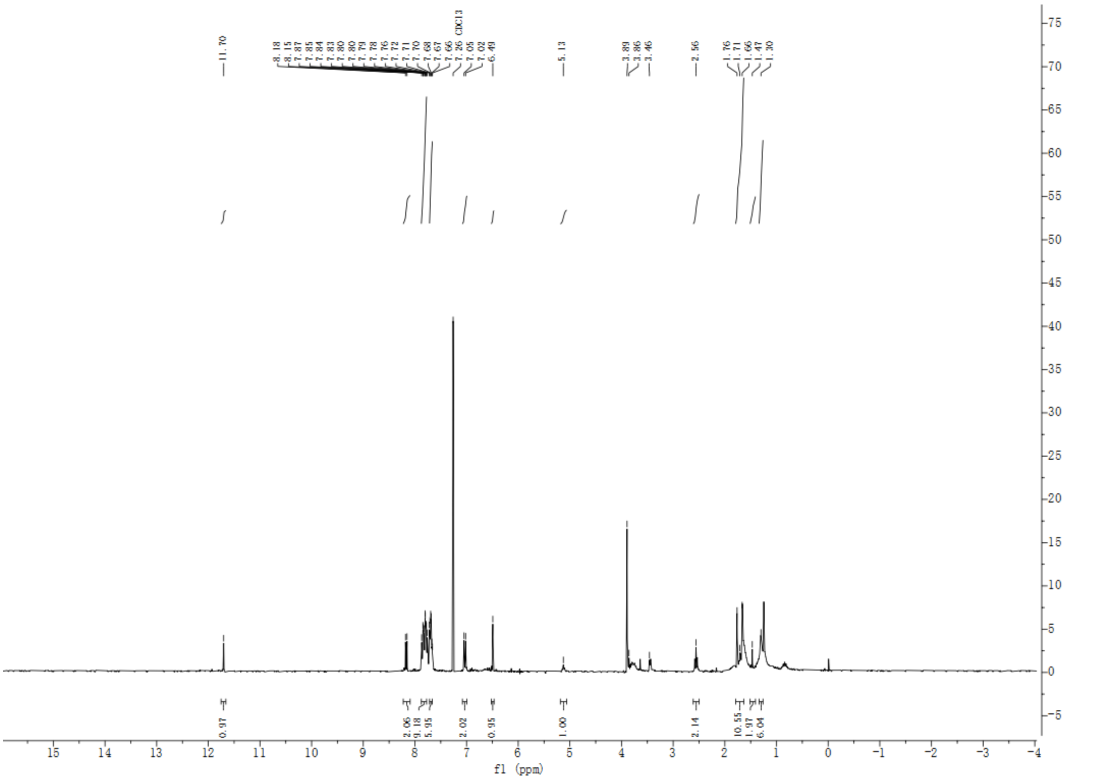

Figure. S11 Compound Mito-ICT-3 ^{13}C NMR (75MHz, $CDCl_3$).

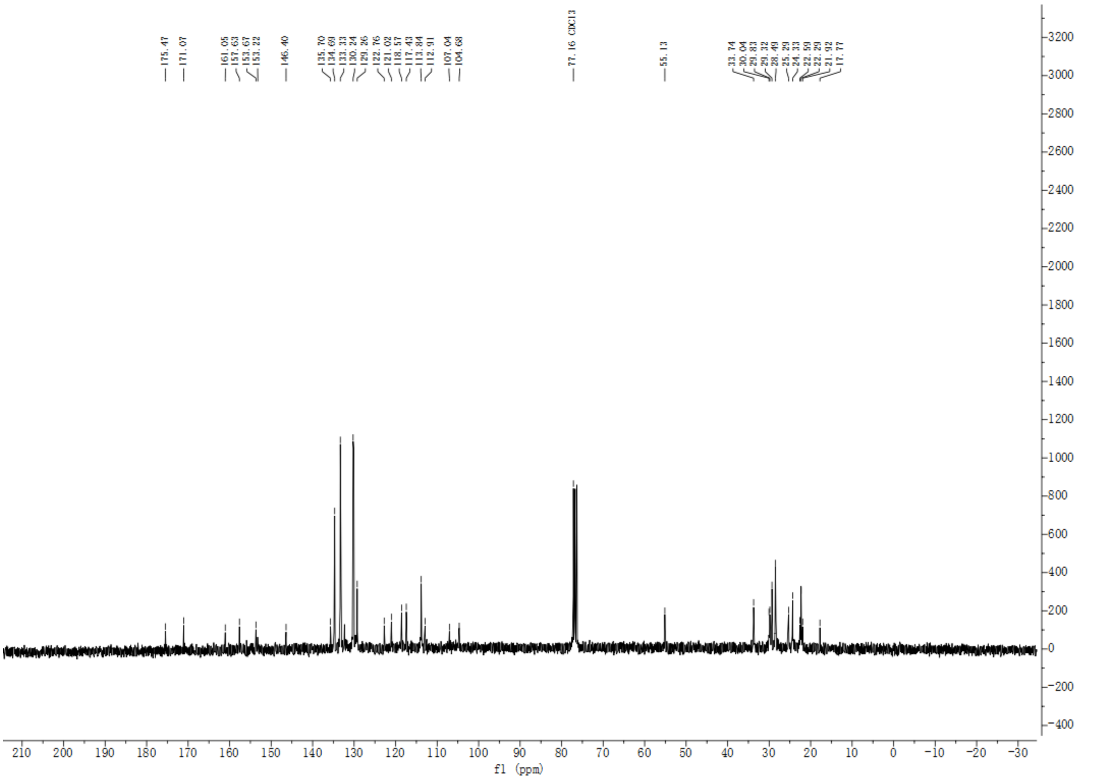

Figure. S12 Compound Mito-ICT-4 ^{1}H NMR (300MHz, $CDCl_3$).

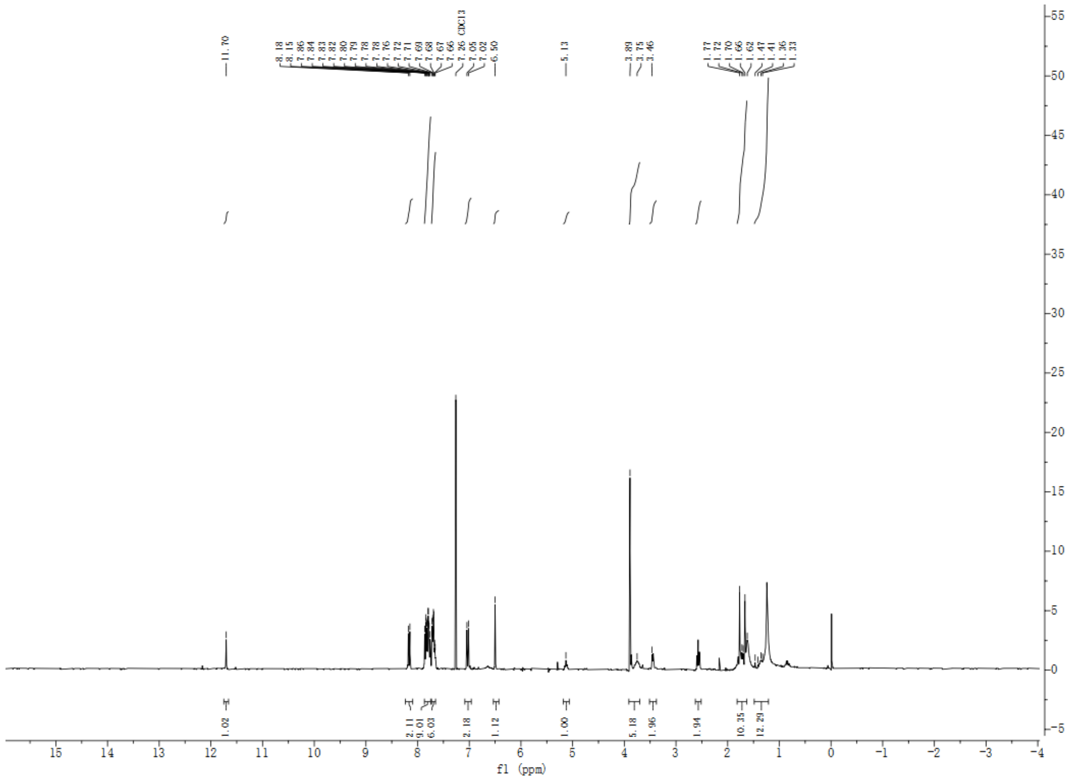

Figure. S12 Compound Mito-ICT-4 ^{13}C NMR (75MHz, $CDCl_3$).

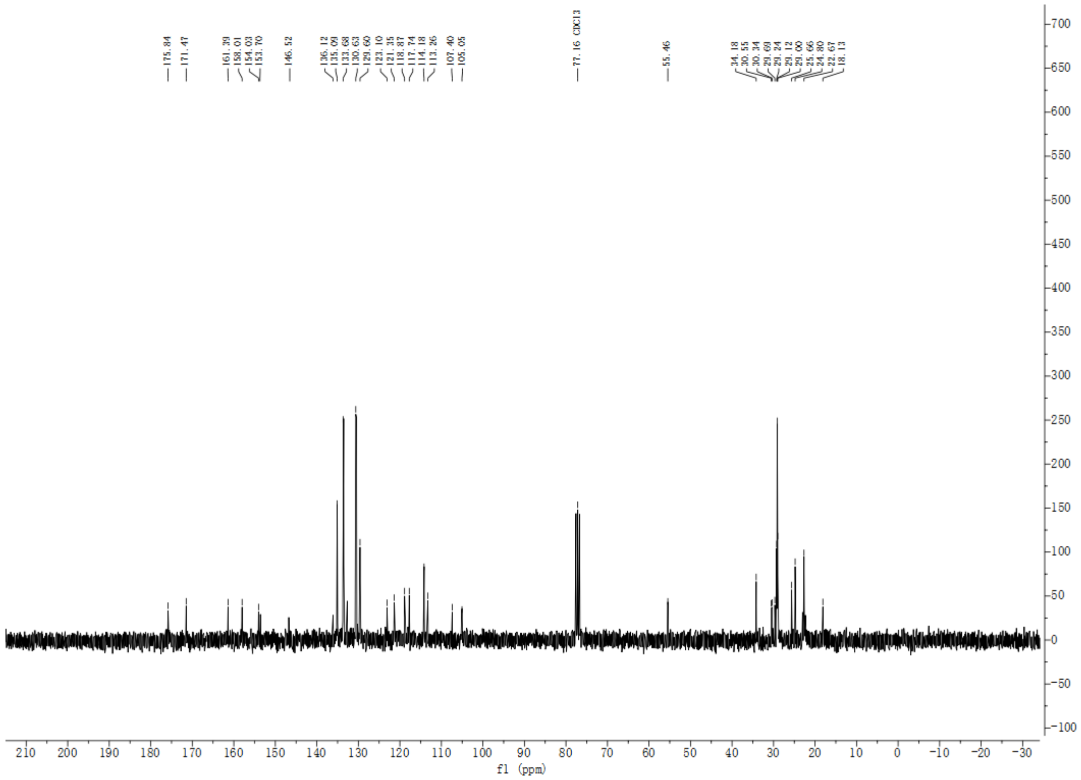

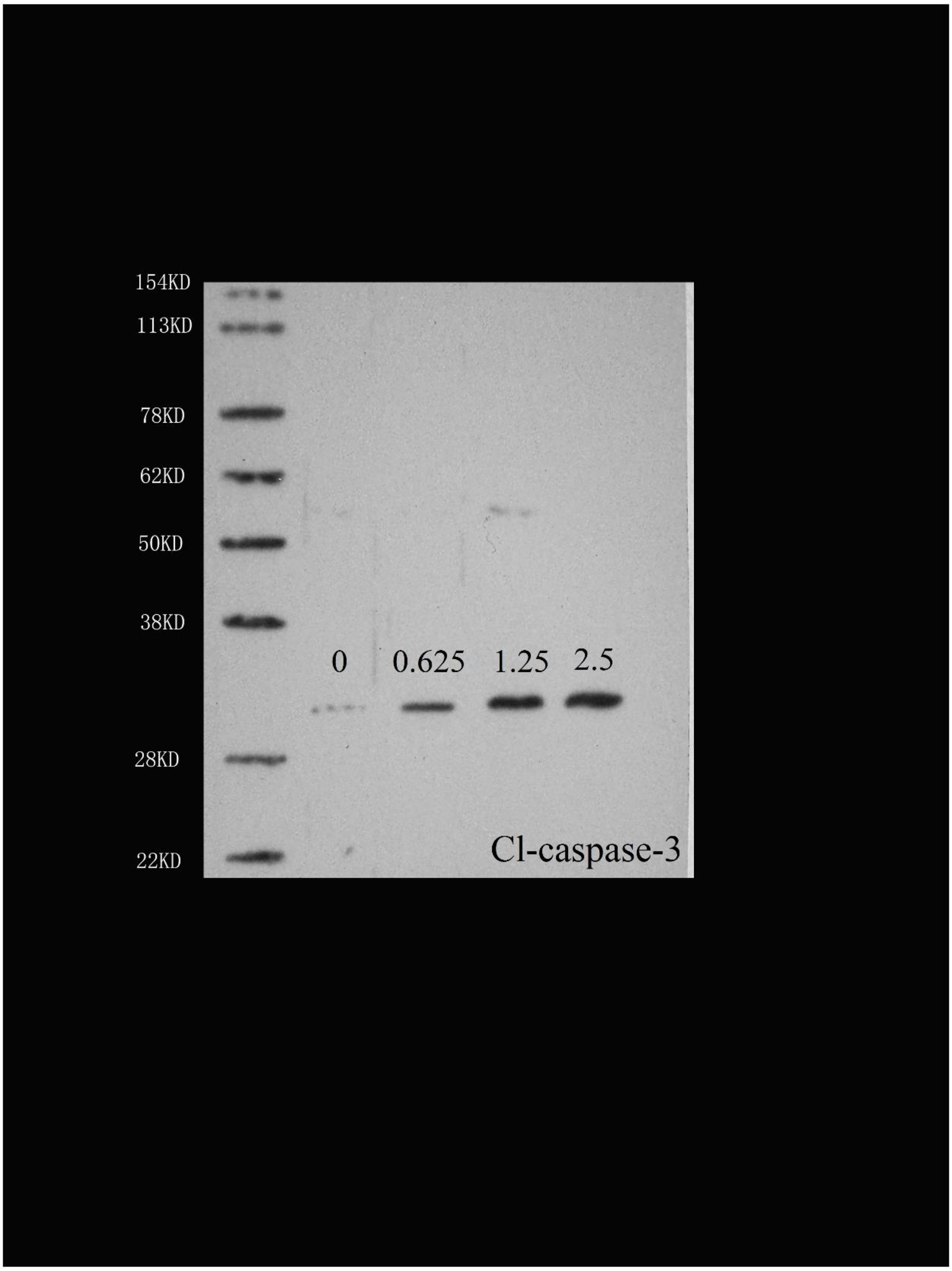

Figure. S13 Effect of Mito-ICT-4 on the level of Cl-caspase-3. BEL-7402 cells were treated with 0.625, 1.25, and 2.5 μM Mito-ICT-4 for 48h.

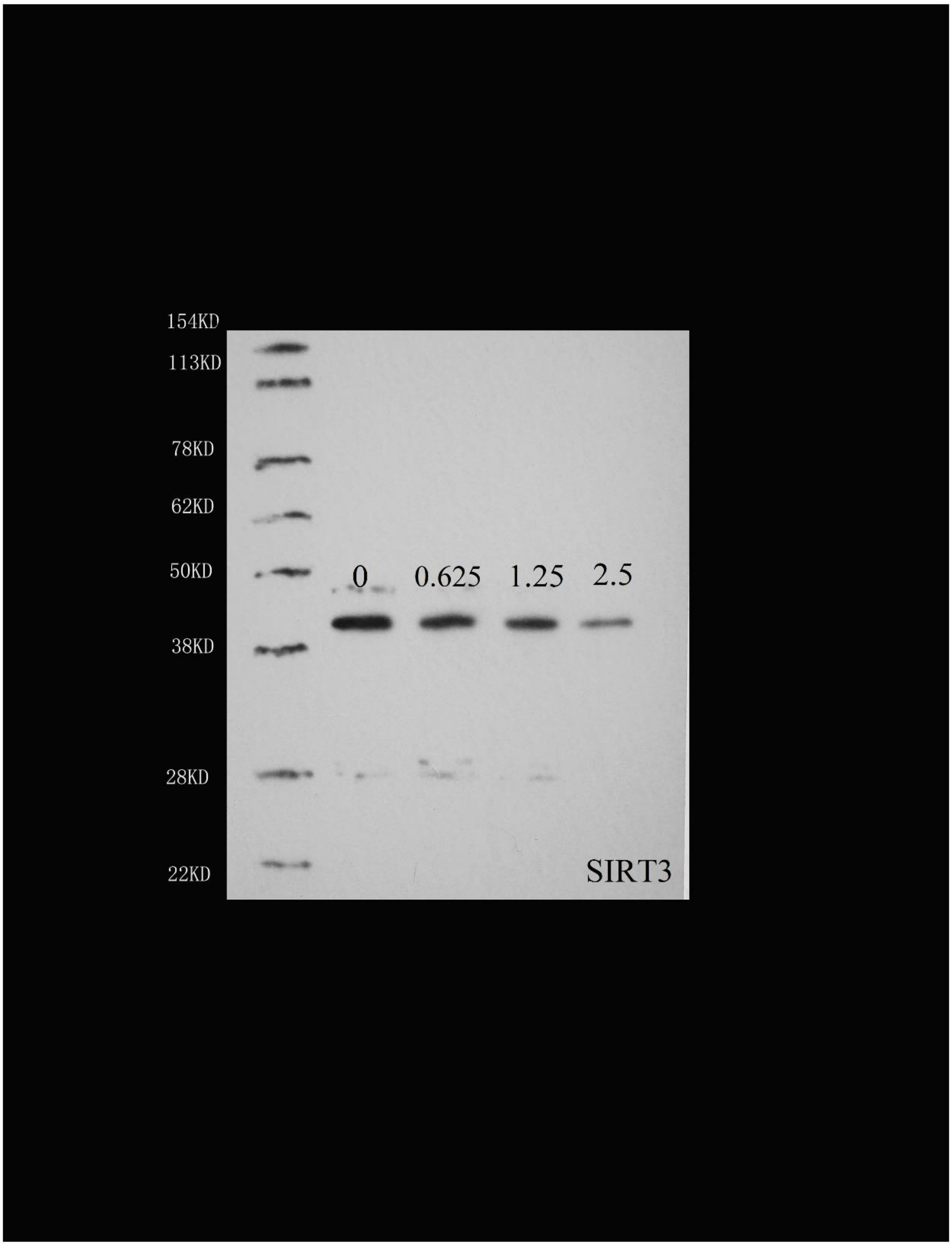

Figure. S14 Effect of Mito-ICT-4 on the level of SIRT3. BEL-7402 cells were treated with 0.625, 1.25, and 2.5 μM Mito-ICT-4 for 48h.

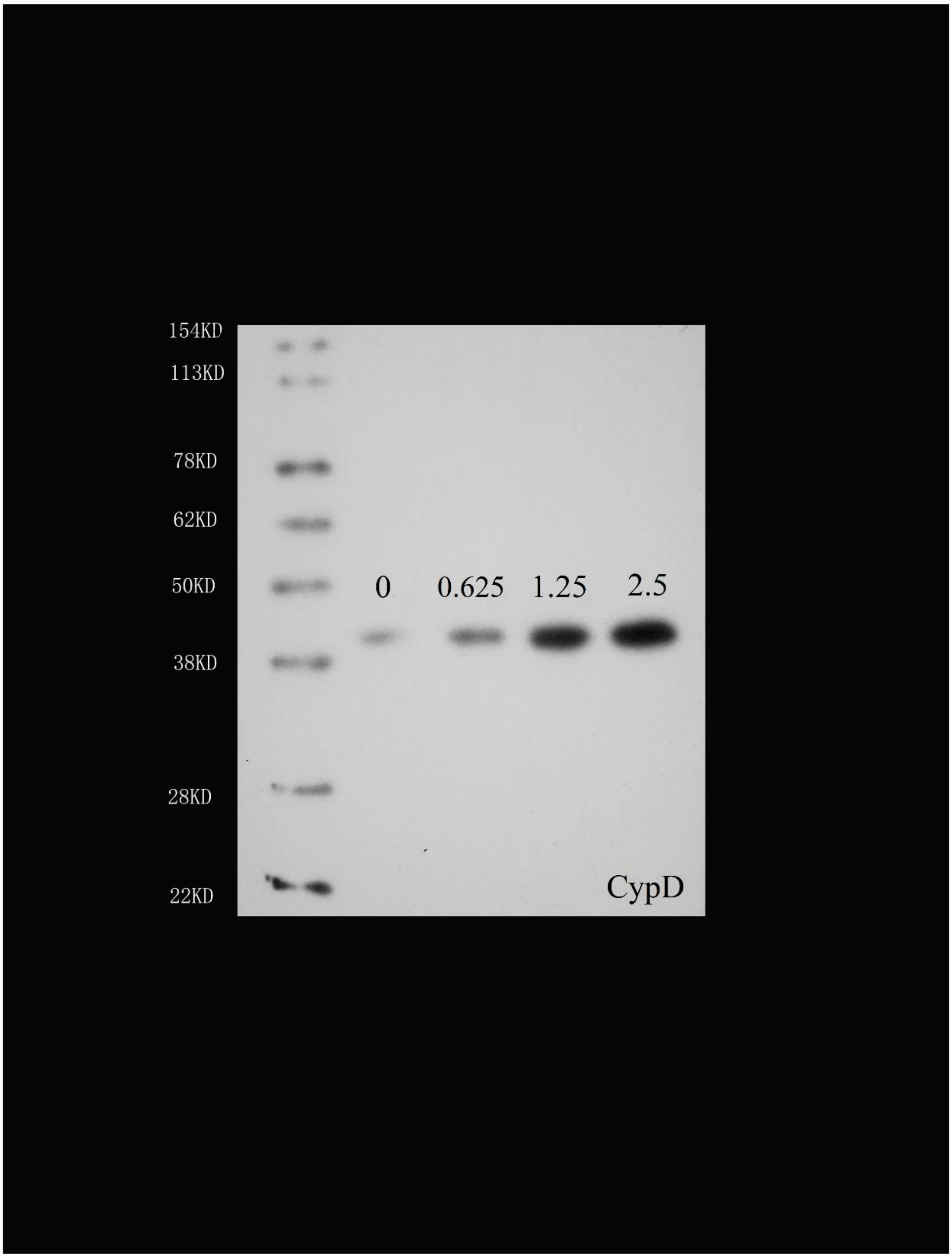

Figure. S15 Effect of Mito-ICT-4 on the level of CypD. BEL-7402 cells were treated with 0.625, 1.25, and 2.5 μM Mito-ICT-4 for 48h.

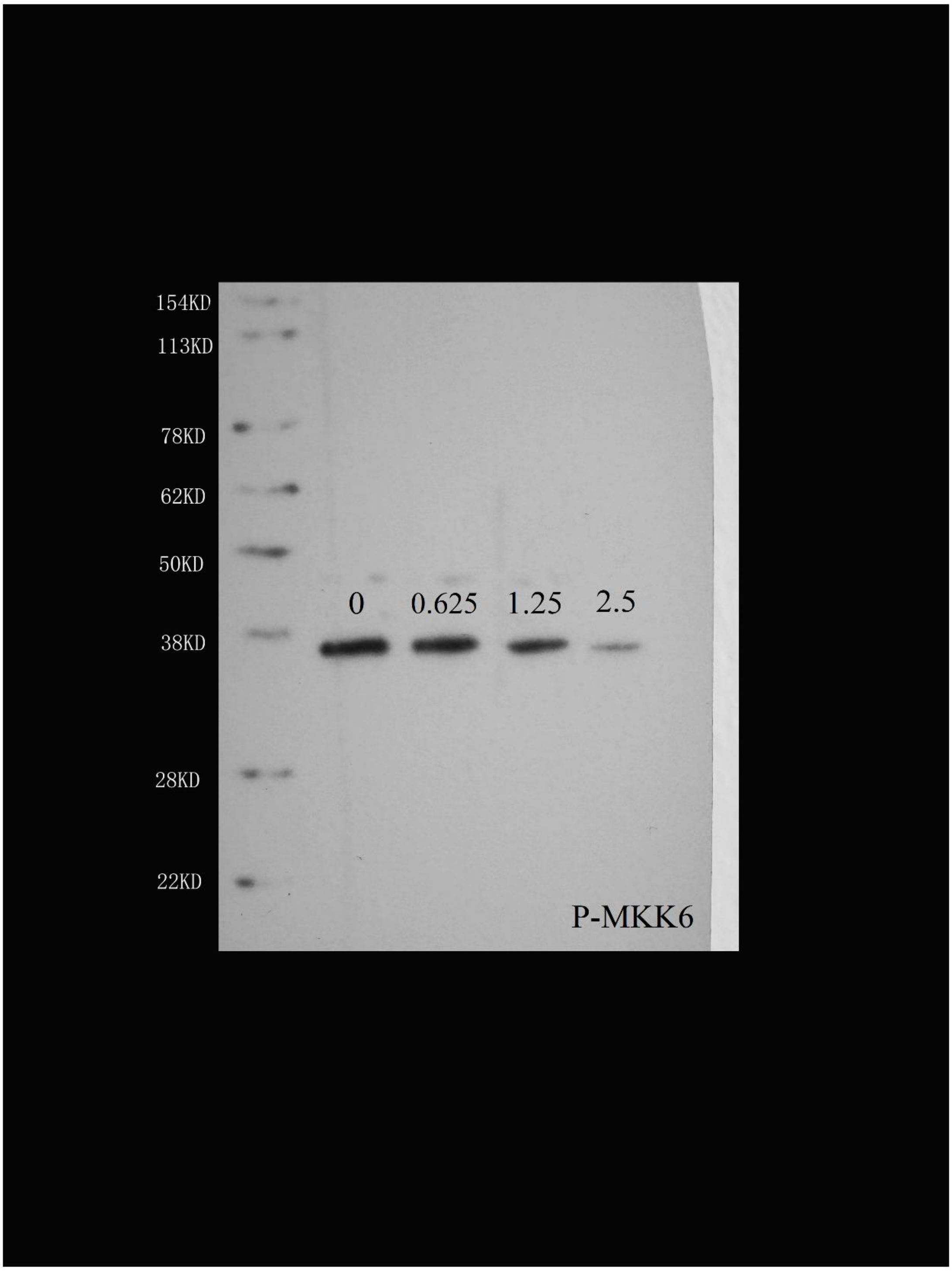

Figure. S16 Effect of Mito-ICT-4 on the level of P-MKK6. BEL-7402 cells were treated with 0.625, 1.25, and 2.5 μM Mito-ICT-4 for 48h.

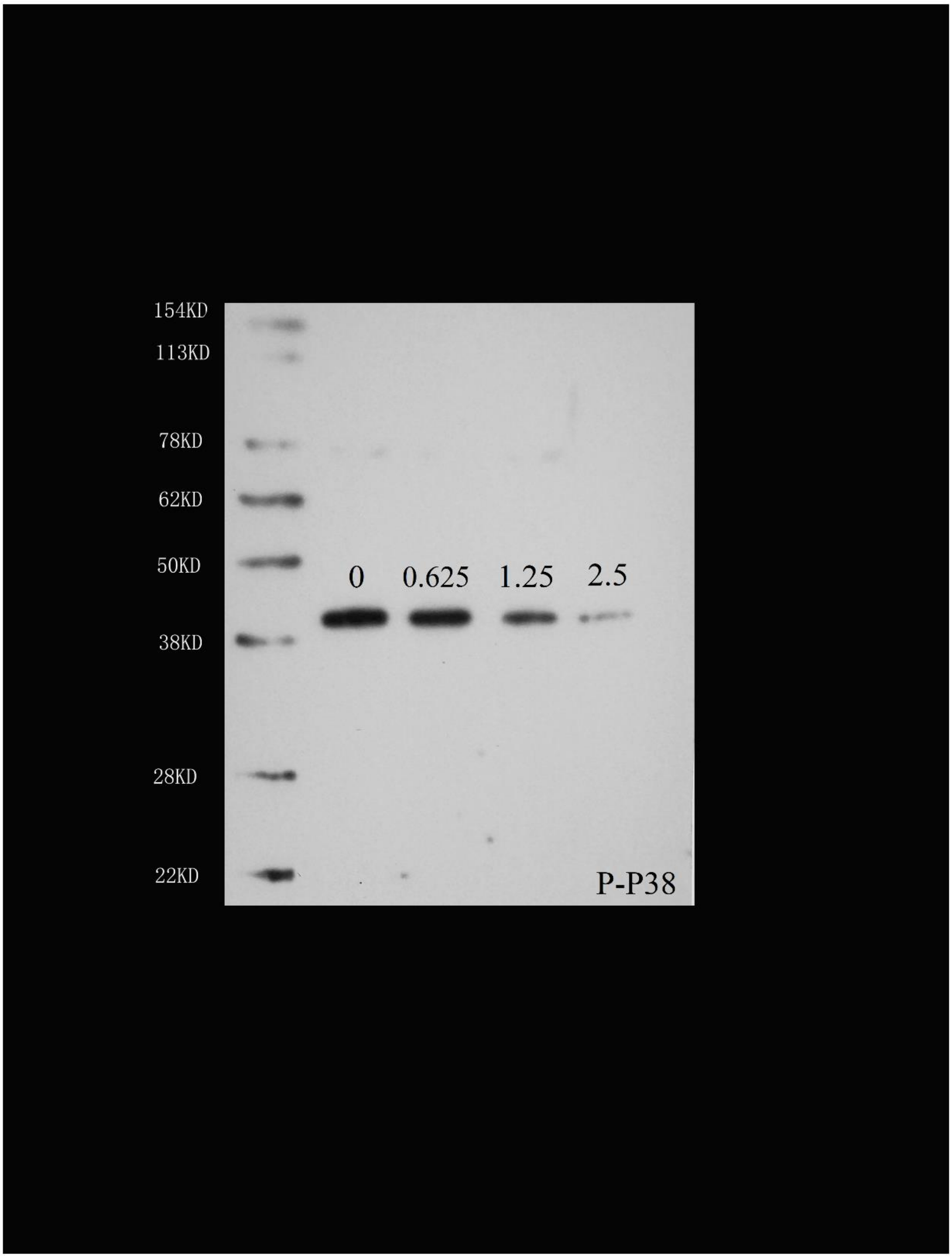

Figure. S17 Effect of Mito-ICT-4 on the level of P-P38. BEL-7402 cells were treated with 0.625, 1.25, and 2.5 μM Mito-ICT-4 for 48h.

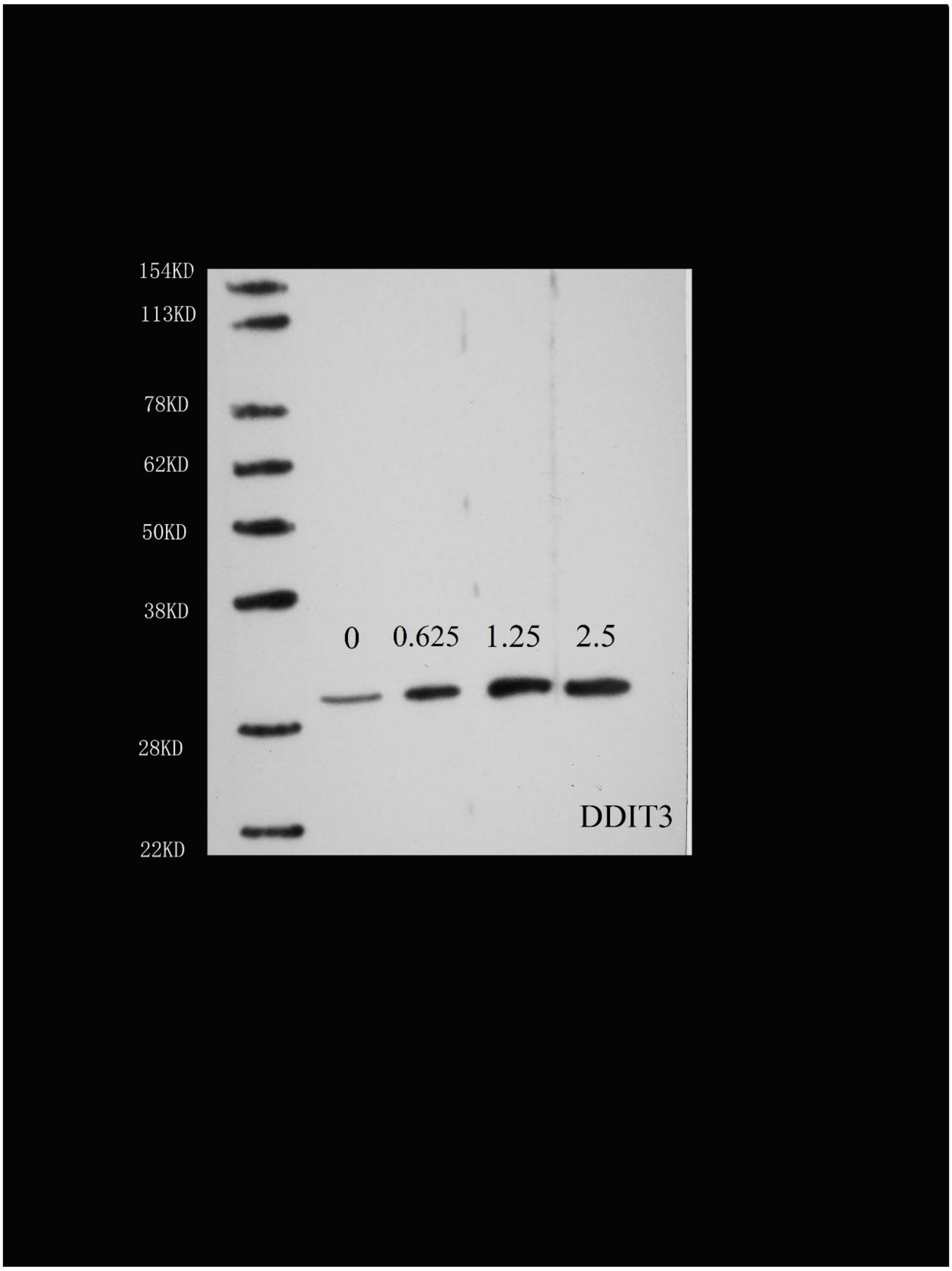

Figure. S18 Effect of Mito-ICT-4 on the level of DDIT3. BEL-7402 cells were treated with 0.625, 1.25, and 2.5 μM Mito-ICT-4 for 48h.

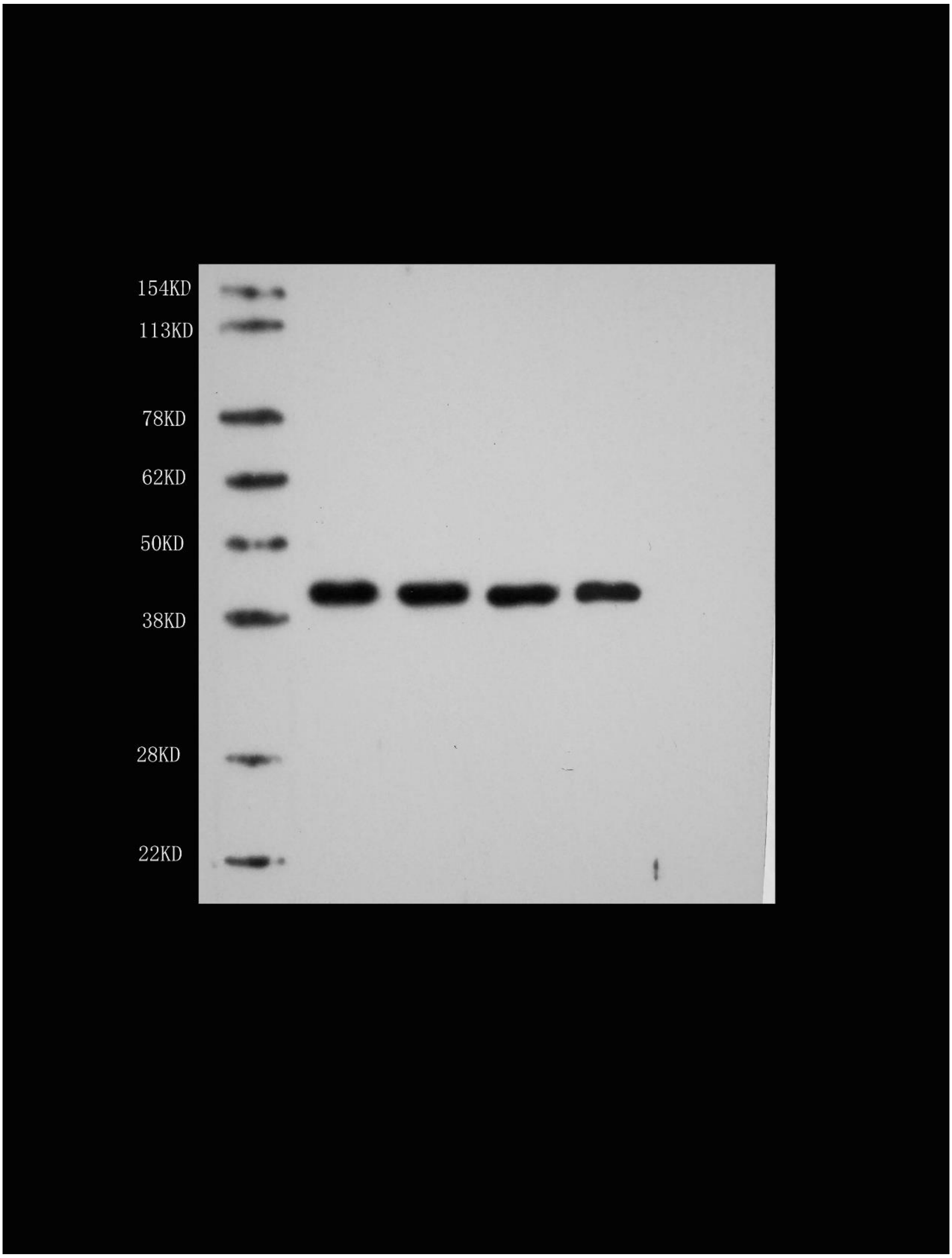

Figure. S19 Internal reference protein β-actin.

Table S1. Real-time fluorescent quantitative PCR primers

Primer name	5′-3′
H-b-actin-F	TAGTTGCGTTACACCCTTTCTTG
H-b-actin-R	TCACCTTCACCGTTCCAGTTT
H-SIRT3-F1	GAGGGTGGTGGTCATGGTG
H-SIRT3-R1	GGGCTTGTAGTTTCCAGGGTA
Cypd-F	ACAGGCGGGAAGTCCATC TACG
Cypd-R	GAACTGGGAGCCGTT GGTGTTAG
MKK6-F	GAGGCTGGCAATTTCAACTAGG
MKK6-R	AATCTCGAGGTGGTGTGGAACT
p38-F	GTTCCCAAATGCTGACTCCA
p38-R	CTCGTCACTCGGGTCGTAAT
DDIT3-F	TGGAACCTGAGGAGAGAGTGTT
DDIT3-R	ACAAGCTCCATGTAGCAAACAG
